# Diversity-Oriented Synthesis and Chemoinformatic Analysis of the Molecular Diversity of sp^3^-Rich Morpholine Peptidomimetics

**DOI:** 10.3389/fchem.2018.00522

**Published:** 2018-10-30

**Authors:** Elena Lenci, Riccardo Innocenti, Gloria Menchi, Andrea Trabocchi

**Affiliations:** Department of Chemistry “Ugo Schiff”, University of Florence, Florence, Italy

**Keywords:** chemical diversity, heterocycles, amino acids, carbohydrates, small molecules, building blocks, spiro-lactam

## Abstract

Diversity-Oriented Synthesis (DOS) consists of generating structurally diverse compounds from a complexity-generating reaction followed by cyclization steps and appendage diversity. DOS has gathered interest to systematically explore the chemical space by generating high-quality small-molecule collections as probes to investigate biological pathways. The generation of heterocycles using amino acid and sugar derivatives as building blocks is a powerful approach to access chemical and geometrical diversity thanks to the high number of stereocenters and the polyfunctionality of such compounds. Our efforts in this field are focused on the generation of diversity-oriented molecules of peptidomimetic nature as a tool addressing protein-protein interactions, taking advantage of amino acid- and sugar-derived polyfunctional building blocks to be applied in couple-pair synthetic approaches. In this paper, the combination of diversity-oriented synthesis and chemoinformatics analysis of chemical space and molecular diversity of heterocyclic peptidomimetics are reported, with particular interest toward carbohydrate- and amino acid-derived morpholine scaffolds with a higher fraction of sp^3^ carbon atoms. Also, the chemoinformatic analysis of chemical space and molecular diversity of 186 morpholine peptidomimetics is outlined.

## Introduction

**Figure 1:**
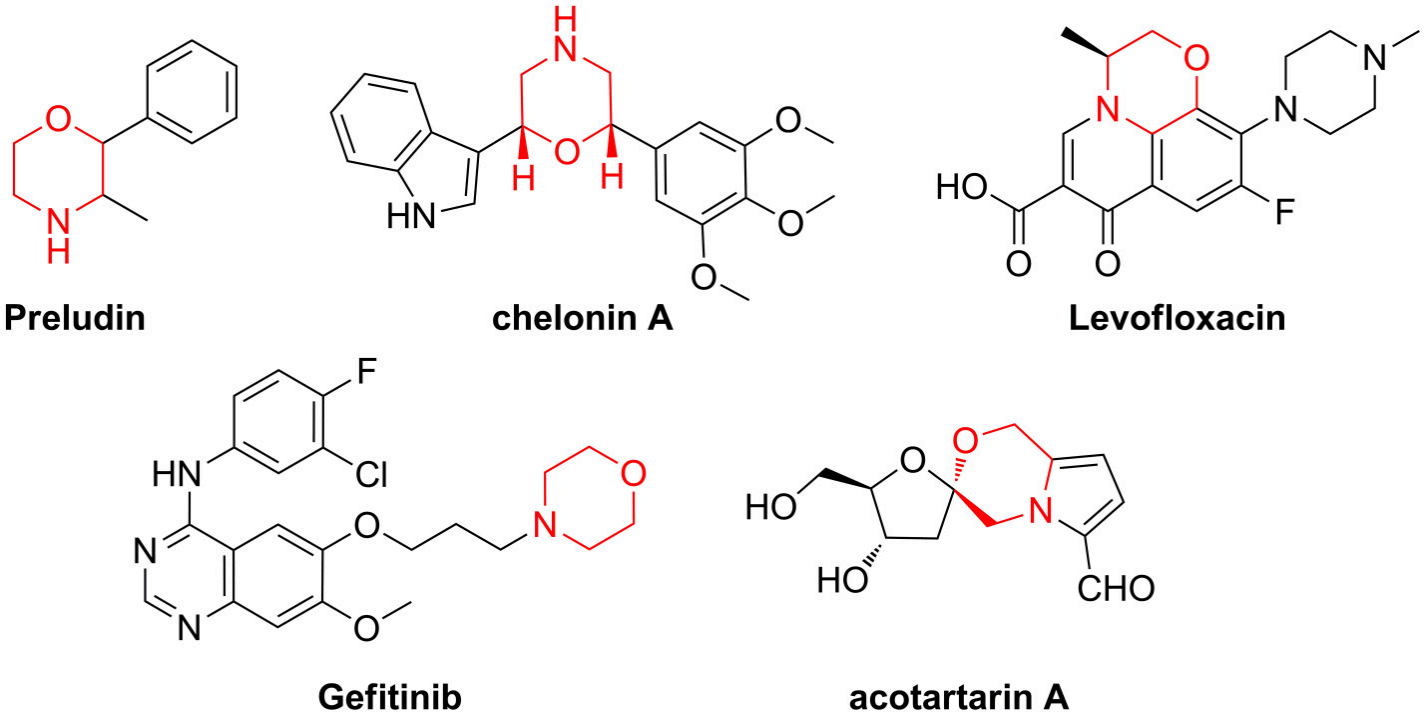
Representative examples of natural products and drugs containing a morpholine ring.

When the molecular targets behind a disease are poorly characterized or difficult to identify, the screening of small-molecule libraries is a powerful starting point for drug discovery programmes (Gerry and Schreiber, 2018). This is especially true considering that many biological mechanisms, such as signal transduction or gene expression, are regulated by protein-protein interaction (PPI), “undruggable” targets that cannot be addressed with existing chemical tools (Wells and McClendon, 2007). Even though many synthetic efforts have given a great advance in improving peptide druggability, this class of compounds covers only 2% of the worldwide drug market (Sun, 2013) and the development of new peptidomimetic scaffolds is still a growing field of medicinal chemistry and chemical biology (Kaminker et al., 2018; Ramaswamy et al., 2018). In this context, Diversity-Oriented Synthesis (DOS) (Trabocchi, 2013; Chauhan et al., 2017; Zeng et al., 2017), where many different molecular scaffolds possessing a high structural complexity are developed using short synthetic strategies, is a convenient approach for the generation of large sets of small molecule peptidomimetics. In particular, in view of creating sp^3^-rich molecular entities, with polyfunctional and stereochemically dense characteristics, building blocks from the chiral pool are increasingly used in DOS, as showed by the relevance recently gained by the biosynthetically inspired divergent approach (Yang et al., 2014; Bender et al., 2018), or the diversity-oriented synthesis of natural-product inspired libraries (Huigens et al., 2013; McLeod et al., 2014; Annamalai et al., 2017; Saleeb et al., 2018). Our efforts in this field are focused on the exploitation of amino acid and sugar derivatives for the generation of peptidomimetic libraries around the morpholine skeleton, as this key nucleus is contained in many natural products and drugs (Figure 1) (Wijtmans et al., 2004; Pal'chikov, 2013).

**Scheme 1:**
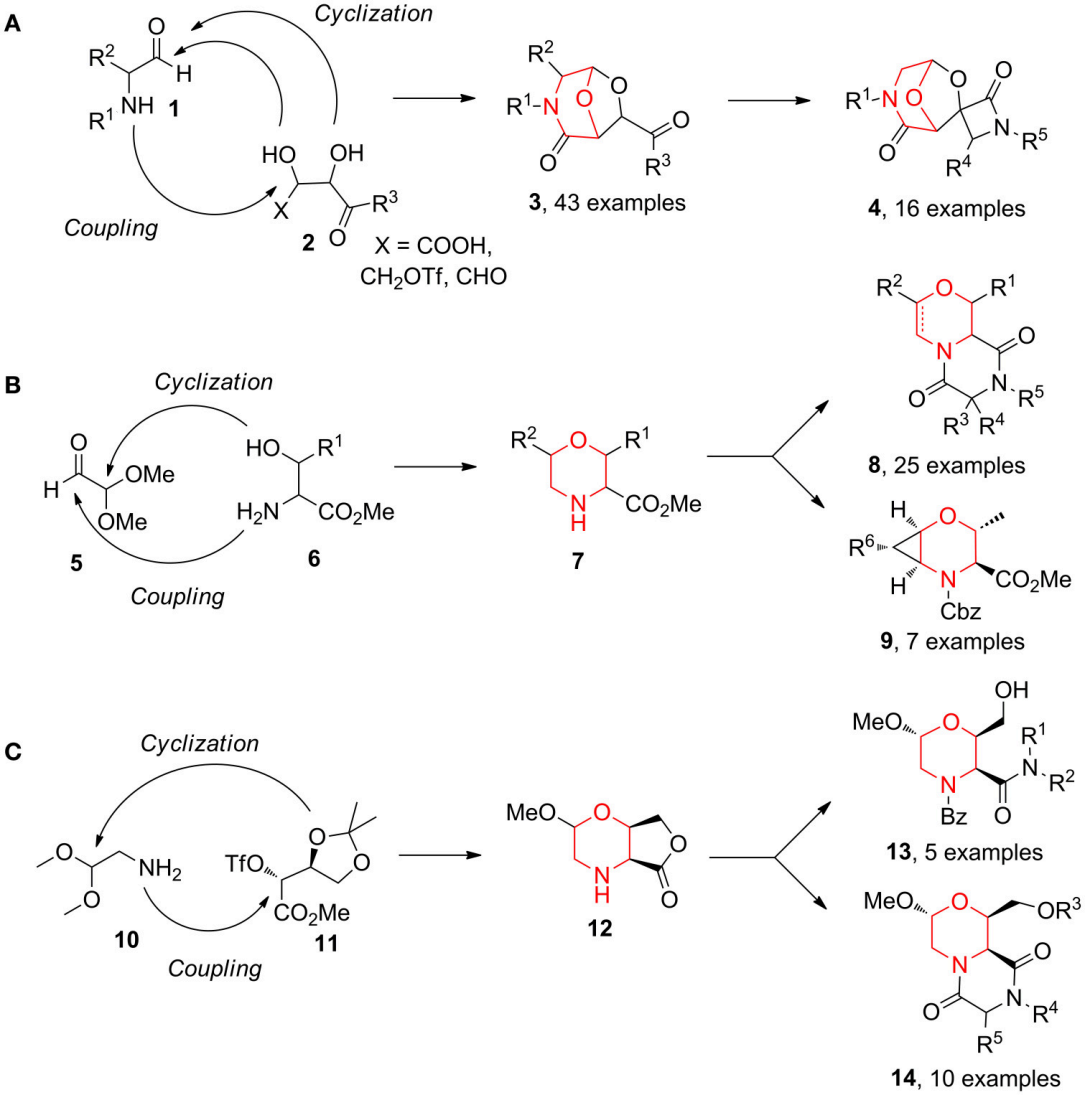
Representative diversity-oriented synthesis of morpholines, starting from amino carbonyl derivative **1** and diol **2 (A)**; from dimethoxyacetaldehyde **5** and amino acid derivative **6 (B)**; and from aminoacetaldehyde **10** and threonate derivative **11 (C)**.

Over the years, we reported the synthesis of many different bicyclic compounds **3** based on the 6,8-dioxa-3-azabicyclo[3.2.1]octane core, an atom-by-atom dipeptide isostere. They proved to be active as aspartyl protease inhibitors (SAP2: Trabocchi et al., 2010; Calugi et al., 2012; HIV: Calugi et al., 2014; BACE1: Innocenti et al., 2017) and as RGD integrin ligands (Cini et al., 2009; Bianchini et al., 2012). The short and convenient synthetic strategies consist of combining two key components from the chiral pool, an amino carbonyl derivative **1** and a diol species **2**, followed by the acid-catalyzed acetalization of the resulting coupling intermediate (Scheme 1A) (Trabocchi et al., 2006). Representative follow-up chemistry was achieved generating spirocylic scaffolds **4** (Trabocchi et al., 2007). This couple/pair approach proved to be even more interesting in a diversity-oriented point of view when α-amino acid derivatives **6** were combined with dimethoxyacetaldehyde **5**, as morpholine acetal scaffold **7** was a good starting point for the generation of many bi- and tricyclic compounds, such as diketopiperazines **8** and 2-oxa-5-azabicyclo[4.1.0]heptanes **9** (Sladojevich et al., 2008; Lalli et al., 2009; Lenci et al., 2015a) (Scheme 1B). Also, bicyclic morpholine lactone **12**, coming from aminoacetaldehyde dimethylacetal **10** and protected methyl threonate derivative **11**, gave structures **13**, taking advantage of lactone aminolysis, and structures **14**, when the aminolysis was combined with diketopiperazine synthesis (Lalli et al., 2009; Ciofi et al., 2010) (Scheme 1C).

**Scheme 2:**
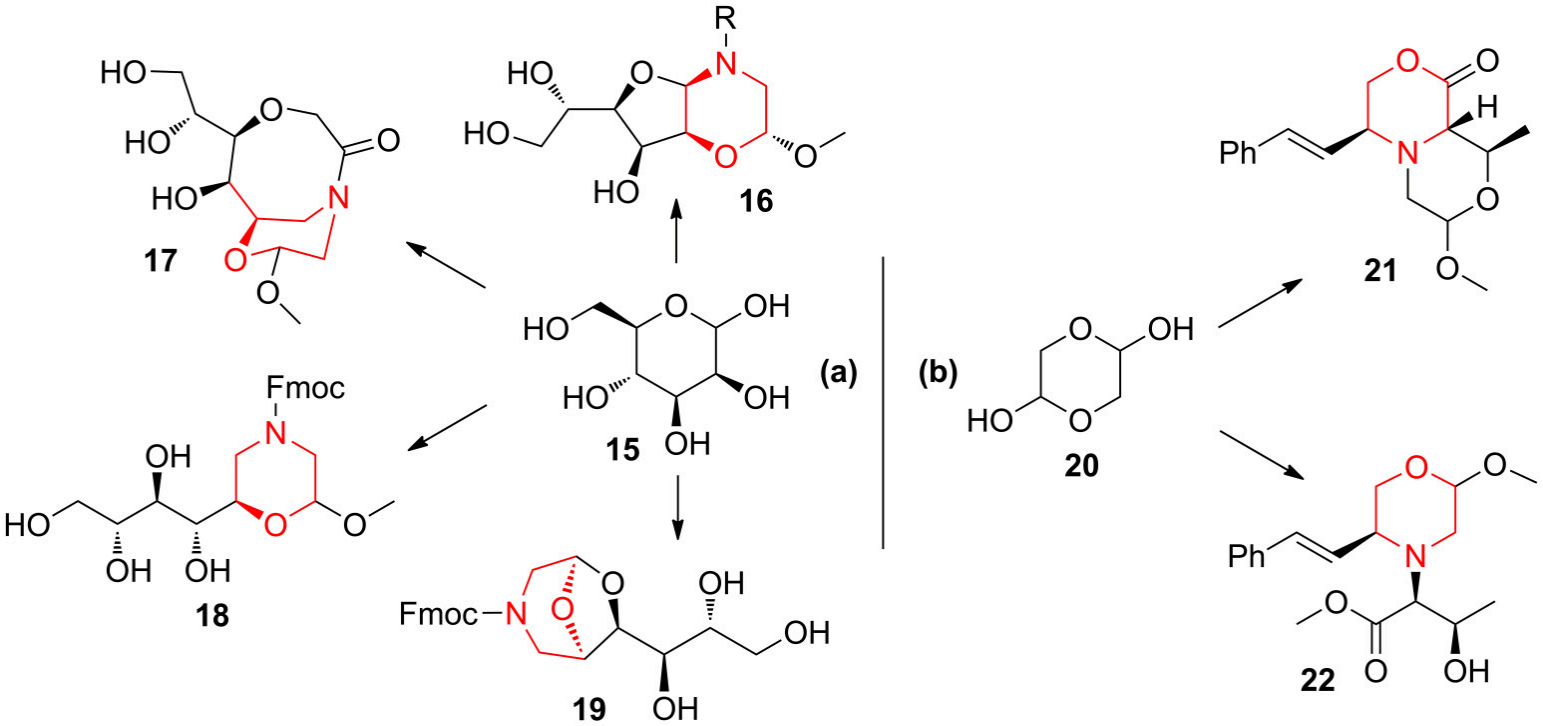
Representative syntheses of morpholine-derived compounds starting from mannose **15 (a)** and from glycolaldehyde **20 (b)**.

Also, the combination of mannose **15** with aminoacetaldehyde **10** allowed to obtain morpholine-derived compounds enriched with polyhydroxylated chains (compounds **16**–**19**, Scheme 2a) exploiting the reactivity of sugar hydroxyl groups toward the acetal moiety (Lenci et al., 2015b, 2016). Similarly, the application of lactone formation and *trans*-acetalization pairing reactions were used in the synthesis of **21**–**22** starting from the Petasis coupling intermediate obtained by glycolaldehyde **20** (Scheme 2b) (Lenci et al., 2017).

**Scheme 3:**
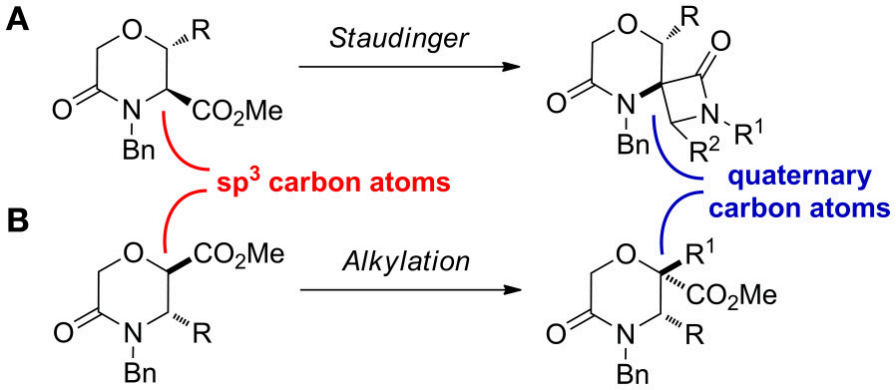
Synthetic approaches exploited to install quaternary carbon atoms on morpholine-3-one. **(A)** the Staudinger ketene-imine reaction on methyl 5-oxomorpholine-3-carboxylate and **(B)** alkylation reaction on methyl 5-oxomorpholine-2-carboxylate.

Considering that a higher scaffold complexity is generally associated with a more successful outcome in drug discovery and development (Clemons et al., 2010; Galloway et al., 2011; Flagstad et al., 2016; Stotani et al., 2016), we recently turned our attention on exploiting the chemistry useful to develop skeletally complex sp^3^-rich morpholines, for example by using multicomponent reactions. In this work, as a further improvement in this direction, we envisioned to install quaternary stereocenters on this nucleus, as they are often present in the structure of many biologically active compounds and pharmaceutical agents (Christoffers and Baro, 2006; Hawner and Alexakis, 2010). This was envisaged by transforming the sp^3^ carbon atom in α-position of the carbomethoxy group of different morpholin-3-one starting materials, by means of the Staudinger reaction, to generate morpholinone-derived spiro-β-lactams (Scheme 3A), and by different alkylation strategies (Scheme 3B).

Finally, the exploration of the chemical space accessed by these new compounds was analyzed using PCA (Principal Component Analysis) and PMI (Principal Moment of Inertia) graphical representation in relation to our in-house library of more than 170 morpholine compounds developed over the years in our laboratory. The entire collection of morpholines was also studied using different chemoinformatic approaches (Colomer et al., 2016) by characterizing the degree of complexity of each library member, by using the Fsp^3^ definition (Lovering et al., 2009), and through the relationship between different drug- and lead-like properties.

## Materials and methods

### Chemistry

Experimental procedures, compound characterization data for newly synthesized compounds **35**–**38** and **42** and NOESY 1D spectra for compounds **37** and **42**, are reported in the Supplementary Material. NMR spectra were collected on a Varian INOVA 400 spectrometer operating at 400 MHz for 1 H. The spectra were obtained in CDCl_3_ solutions. Proton signals were assigned via TOCSY spectra, and NOESY spectra provided the data used in the conformational analyses. TOCSY spectra were recorded with 2,048 points in t1, 200 points in t2, and 8 scans per t2 increment, and 80 ms as mixing time. NOESY spectra were recorded with a similar number of t1 and t2 points unless otherwise noted, 32 per t2 increment, and 500 ms as mixing time. 1D NOESY experiments were carried out using 64 increments and 500 ms as mixing time.

### Molecular modeling methods

Molecular modeling calculations were carried out on compounds **35**–**38** and compound **42** so as to assess the global minimum conformer and to gain insight into the detailed structure of the molecular scaffolds. Energy-minimized conformations of **35**–**38** and compound **42** were achieved using SPARTAN Version 5.1 (Wavefunction, Inc., Irvine, C). Conformational searches were carried out using Monte Carlo method within MMFF94 force field (Halgren, 1996) and the AM1 semiempirical method (Dewar et al., 1985) was used to optimize the global minimum conformer.

### Chemoinformatics analysis

#### PCA analysis

The web-based public tool ChemGPS-NP (http://chemgps.bmc.uu.se/) was used for the PCA analysis of compounds **35**–**38** and compound **42**, to compare their chemical properties with those of an in-house library of morpholine-derived compounds. ChemGPS-NP can be applied for comprehensive chemical space navigation and exploration in terms of global mapping on to a consistent 8-dimensional map of structural characteristics. The first four dimensions of the ChemGPS-NP map capture 77% of data variance. Chemical compounds were positioned onto this map using interpolation in terms of PCA score prediction. SMILES codes for all compounds were retrieved using ChemBioDraw Ultra 12.0 and submitted to ChemGPS-NP for achieving the corresponding PC scores. The PCA data were then used for the construction of PC1 (representing size, shape, and polarizability) vs. PC2 (representing aromatic and conjugation related properties).

#### PMI analysis

Principal moments of inertia analysis was carried out by calculating the lowest energy conformation of compounds **35**–**38** and compound **42**, and each compound from an in-house library of morpholine-derived compounds. The conformational search was performed using the built-in AMMP molecular mechanics algorithm with default parameters of the VEGA ZZ molecular modeling software package v.3.0.1 (Pedretti et al., 2002). Once the lowest energy conformer was calculated, the three principal moments of inertia (Ixx, Iyy, Izz) and the normalized principal moments of inertia were determined. Specifically, the three calculated principal moments of inertia were sorted by ascending magnitude I_1_, I_2_, and I_3_. Subsequently, in order to eliminate completely the dependency of the chosen representation on the size of the molecules, normalization was performed by dividing the two lower PMI-values (I_1_ and I_2_) by the highest value (I_3_), generating two characteristic values of normalized PMI ratios (NPRs) for each compound (I_1_/I_3_ and I_2_/I_3_). Then, NPR1 (I_1_/I_3_) and NPR2 (I_2_/I_3_) were plotted on a triangular graph with the vertices (0,1), (0.5,0.5), and (1,1) representing a perfect rod, disc and sphere, respectively.

#### Calculation of medicinally-relevant molecular properties

Molecular weight, cLogP, and the number of sp^3^ carbon atoms, stereogenic centers, rotatable bonds, hydrogen bond acceptors and donors were calculated using the web-based public tool FAFDrugs (Free ADME-Tox Filtering Tool), developed at the Paris Diderot University (Lagorce et al., 2015). LogP values are computed by using the xLogP3 program (Cheng et al., 2007), enhanced by employing an in-house library of experimental logP-values from the PHYSPROP database (Lobell et al., 2006) as several models showed that xLogP3 and cLogP methods give similar results (Mannhold et al., 2009). Fsp^3^ was calculated as the number of sp^3^ hybridized carbon atoms vs. the total carbon count. FC^*^ was calculated as the number of stereocenters vs. the total carbon count. Rotatable bonds were defined as any single bond, not in a ring, bound to a non-terminal heavy (i.e., non-hydrogen) atom, excluding amide C-N bonds. Hydrogen bond donors were taken as the sum of all OHs and NHs, and hydrogen bond acceptors were taken as the sum of all oxygen and nitrogen atoms without a formal positive charge, excluding pyrrole nitrogen, heteroaromatic oxygen and higher oxidation states of nitrogen, in agreement with the Lipinski definition (Lipinski, 1997).

## Results and discussion

### Synthesis

**Scheme 4:**
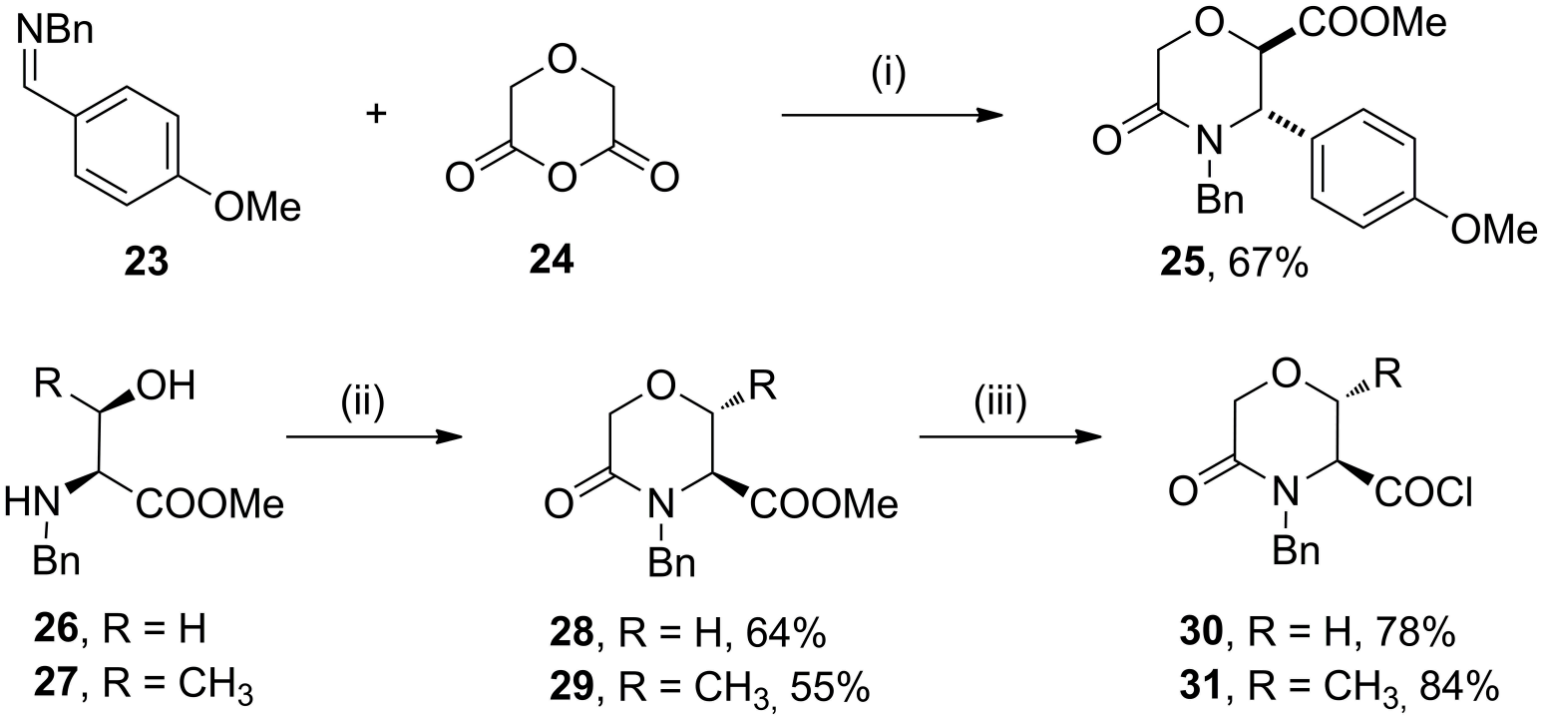
Synthesis of methyl 5-oxomorpholine-2-carboxylate **25** and methyl 5-oxomorpholine-3-carboxylates **28** and **29** and preparation of acyl chloride derivatives **30** and **31**. Reagents and conditions: (i) dry toluene, 80°C, 4 h; then SOCl_2_, MeOH, reflux, 2 h; (ii) BrCOCH_2_Br, Et_3_N, dry CH_2_Cl_2_, −15°C, 1 h; then NaH, THF dry, 0 °C - r. t., 1 h; (iii) LiOH, H_2_O/THF, r. t., 16 h; then SOCl_2_, reflux, 2 h.

As case study to install quaternary stereocenters on the morpholine nucleus, we explored simple synthetic methodologies capable of transforming the sp^3^ carbon atom in the α-position of the carbomethoxy group of different morpholin-3-one compounds. In particular, we selected methyl 5-oxomorpholine-2-carboxylate **25** derived by the application of the Castagnoli-Cushman reaction (Dar'in et al., 2015) between imine **23** and 1,4-dioxane-2,6-dione (**24**), and methyl 5-oxomorpholine-3-carboxylates **28** and **29**, obtained respectively from serine and threonine derivatives **26**-**27** after the acylation with α-bromoacetylbromide and subsequent NaH-mediated intramolecular cyclization reaction (Scheme 4). To improve the scaffold complexity and to install quaternary stereocenters on these compounds, we firstly studied the Staudinger reaction (Alcaide et al., 2007; Cossío et al., 2008; Omidvari and Zarei, 2018) with different aromatic imines to generate polycyclic spiro-β-lactams, in agreement with previous studies on 3-aza-6,8-dioxabicyclo[3.2.1]octane bicycles giving compounds **4** (Trabocchi et al., 2007). In particular, compounds **28** and **29** were transformed into the more reactive acyl chloride derivatives **30**–**31** in order to generate the intermediate ketene more easily and to avoid the formation of amide by-products (Scheme 4).

However, after refluxing the acyl chloride **30**-**31** in the presence of triethylamine as a base and aromatic imines **32**–**34** in toluene for 16 h, the spiro-β-lactams **35**–**38**, characterized by the 8-oxa-2,5-diazaspiro[3.5]nonane-1,6-dione molecular framework, were obtained in moderate yields, as a consequence of the formation of amide by-products **39**–**41** (Table 1).

**Table 1:** Synthesis of spiro-β-lactams **35**–**38** from serine and threonine-derived morpholine derivatives **30** and **31**.

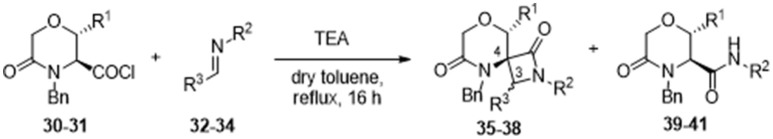						
**Entry**	**Imine**	**R**^1^	**R**^2^	**R**^3^	**Yield (Product)**	**Diastereomer**
1	**32**	H	CH_2_Ph	Ph	52% (**35**) + 25% (**39**)	(3,4)-*cis*
2	**33**	H	*p*-CH_3_Ph	*p*-OMePh	35% (**36**) + 33% (**40**)	3:1 (3,4)-*cis*/ (3,4)-*trans*
3	**33**	CH_3_	*p*-CH_3_Ph	*p*-OMePh	–	–
4	**32**	CH_3_	CH_2_Ph	Ph	15% (**37**)	(3,4)-*cis*
5	**34**	CH_3_	CH_2_Ph	*p*-OMePh	19% (**38**) + 11% (**41**)	(3,4)-*cis*

Considering that the nucleophilicity of the amine derivatives comprising the imine proved to affect the yields, only aromatic imines were taken into account. Also, as shown in Table 1, the steric hindrance of both imine and morpholine counterparts resulted in reducing drastically the yield. In particular, best results were obtained starting from serine-derived morpholine **30** using *N*-benzylidene-1-phenylmethanamine **32** and *N*-(4-methoxybenzylidene)-4-methylaniline **33**, even though the higher steric hindrance of this second imine resulted in the achievement of compound **36** in lower yield (35% instead of 52%, Table 1, entry 1 and 2). On the other hand, threonine-derived morpholine **31** was found to be less reactive and unstable, as a consequence of the presence of the methyl group adjacent to the ketene functionality. In fact, no reaction was observed with imine **33** (Table 1, entry 3), whereas the use of *N*-benzylidene-1-phenylmethanamine **32** and *N*-(4-methoxybenzylidene)-4-phenylmethanamine **34** yielded the spiro compounds **37** and **38** in low yields (Table 1, entry 4 and 5, respectively) and with many degradation products, confirming the difficulty in achieving highly substituted spiro-β-lactams, as also reported (Bari and Bhalla, 2010).

**Figure 2:**
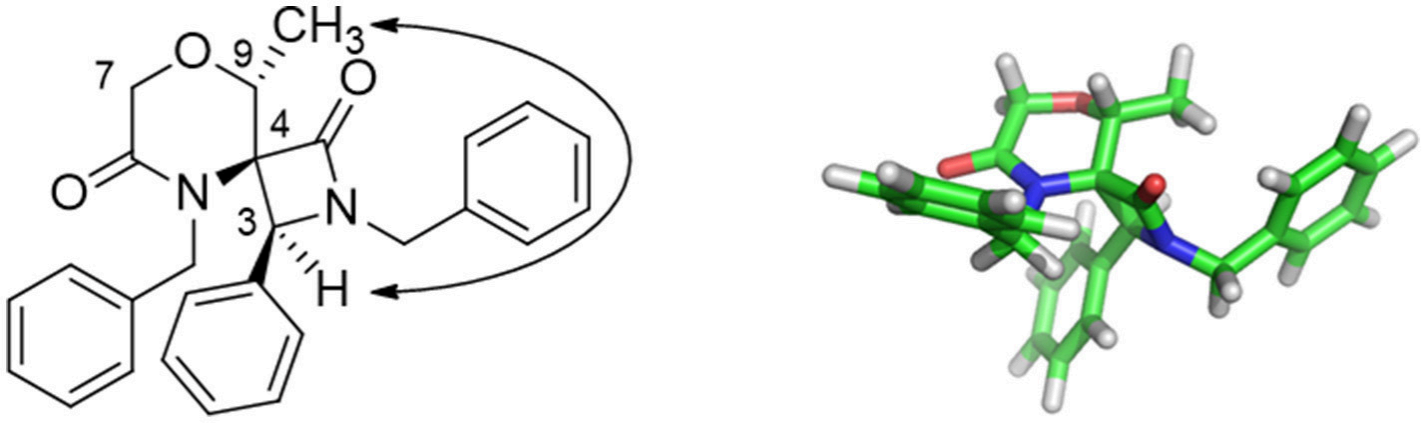
Key NOESY peak **(Left)**, and lowest energy conformer **(Right)** of compound **37** showing the major stereoisomer resulting from the two newly-generated stereocenters at C-3 and C-4.

Nevertheless, interesting results were obtained as regarding the diastereoselectivity. In fact, despite the four theoretically possible diastereomers, in all cases the *cis*-products were obtained as a major or single stereoisomer, as shown by 1D and 2D NOESY experiments carried out on spiro compound **37** and **35** (see Figures S13, S14). In particular, the existence of a NOESY peak between H-3 and the methyl group at C-9 for compound **37** proved the relative configuration as reported in Figure 2. The absence of any correlation between the methyl group and H-7 suggested that the methyl group is oriented in equatorial position. Although purely indicative, this observation was found to be reasonable for such a constrained structure and was in agreement with the global minimum conformer resulting from molecular modeling calculations (Figure 2, right). Specifically, the calculated distance between H-3 and the CH_3_ atoms was 2.1 Å, whereas for the other possible diastereomer at the spiro position this distance was found being more than 4 Å. Similar structural arrangement was ascertained for compound **35**, with the C-1 carbonyl group pointing toward C-9 and the H-3 showing a strong NOESY correlation with H-9 protons, whereas the same *cis*-configuration was evinced for the other compounds by comparing the diagnostic signal of the H-3 proton, which appeared as a singlet in an unambiguous region of ^1^H-NMR spectrum between 4.74 and 4.84 ppm. This diastereoselectivity is in agreement with what observed for similar spiro-β-lactams obtained starting from proline-derived ketenes (Khasanov et al., 2004) and 6,8-dioxabicyclo[3.2.1]octane-derived ketenes (Trabocchi et al., 2007), as the widely accepted mechanism of the reaction involves the nucleophilic attack of the imine on the ketene species to give a zwitterionic intermediate, which preferentially undergoes an outward conrotatory ring closure, due to stabilizing stereoelectronic effects.

**Scheme 5:**
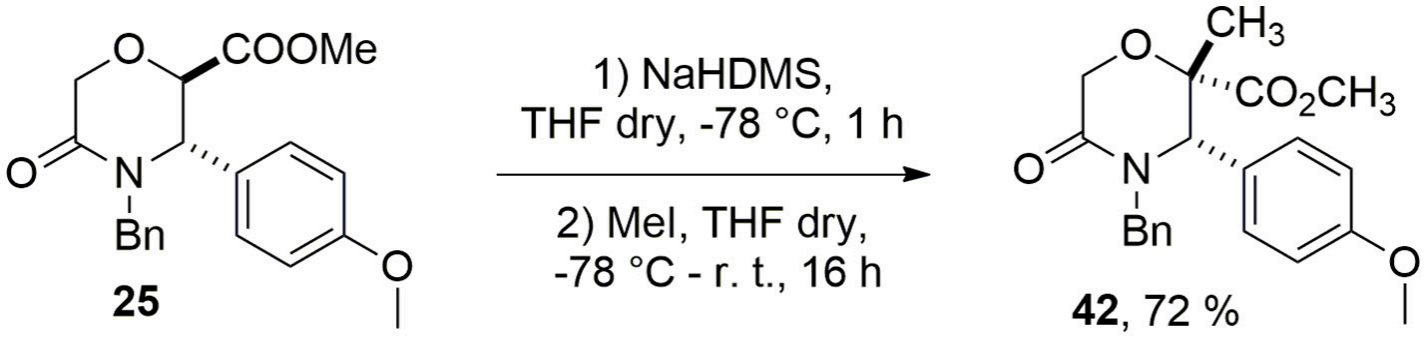
Methylation of 5-oxomorpholine-2-carboxylate **25**.

Unfortunately, when the Staudinger reaction was performed between the acid chloride of methyl 5-oxomorpholine-2-carboxylate **25** and aromatic imines **32**–**34**, only degradation products were observed. Thus, in order to install a quaternary stereocenter on this morpholin-3-one, we explored a complementary approach based on an alkylation strategy, and in particular, as a case study, we performed the methylation of the α-carbon of the carbomethoxy group of **25** using NaHDMS as a strong base to generate the intermediate carbanion (Scheme 5).

**Figure 3:**
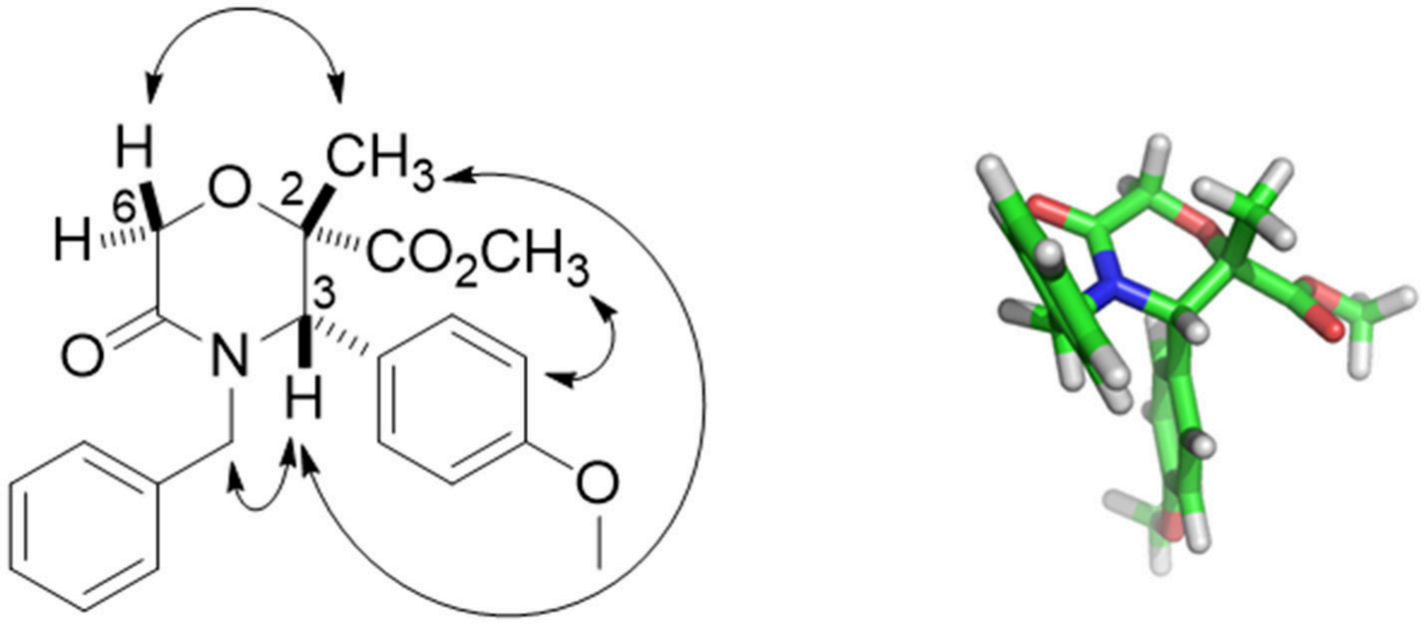
Key NOESY peaks **(Left)**, and lowest energy conformer **(Right)** of compound **42** showing the aryl and methyl groups in axial positions.

Compound **42** was obtained with 72% yield as a single stereoisomer, showing inversion of the configuration at the α-carbon. Structure analysis performed by NMR and molecular modeling calculations showed a half-chair conformation for the morpholinone scaffold possessing both the methyl and aryl groups in axial position and with a *trans* geometry. Specifically, the *trans* arrangement was ascertained by key NOESY peaks between H-3 and CH_3_ at C-2, and a strong NOESY interaction between H-6 and the methyl group at C-2, suggesting the methyl group being positioned in axial orientation (Figure 3).

### Chemoinformatic analysis

**Figure 4:**
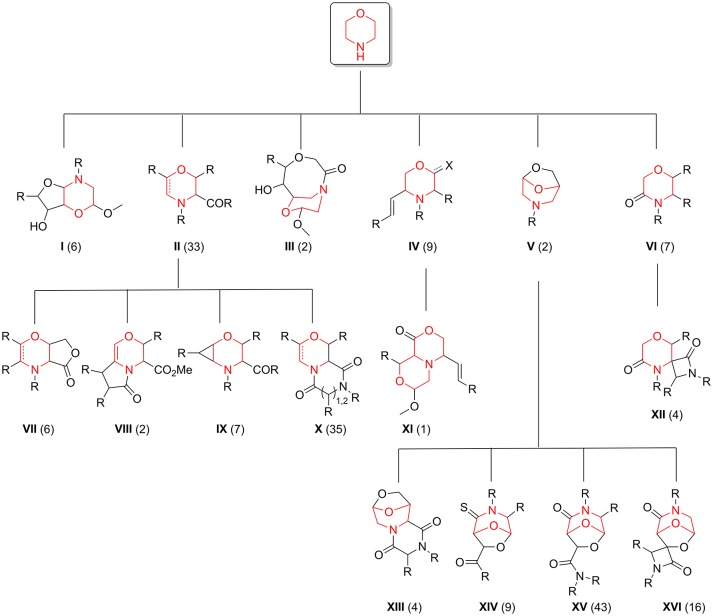
Morpholine-containing molecular scaffolds of our in-house library. The number of compounds in the library representing each scaffold is displayed in brackets.

The exploration of the chemical space accessed by newly synthesized compounds **35**–**42**, in relation to the pool of 176 morpholine-derived small molecules previously synthesized in our laboratories, was then studied by using different chemoinformatic approaches (see Figure 4 for a scaffold tree composed by all the 16 different molecular frameworks present in this library).

**Figure 5:**
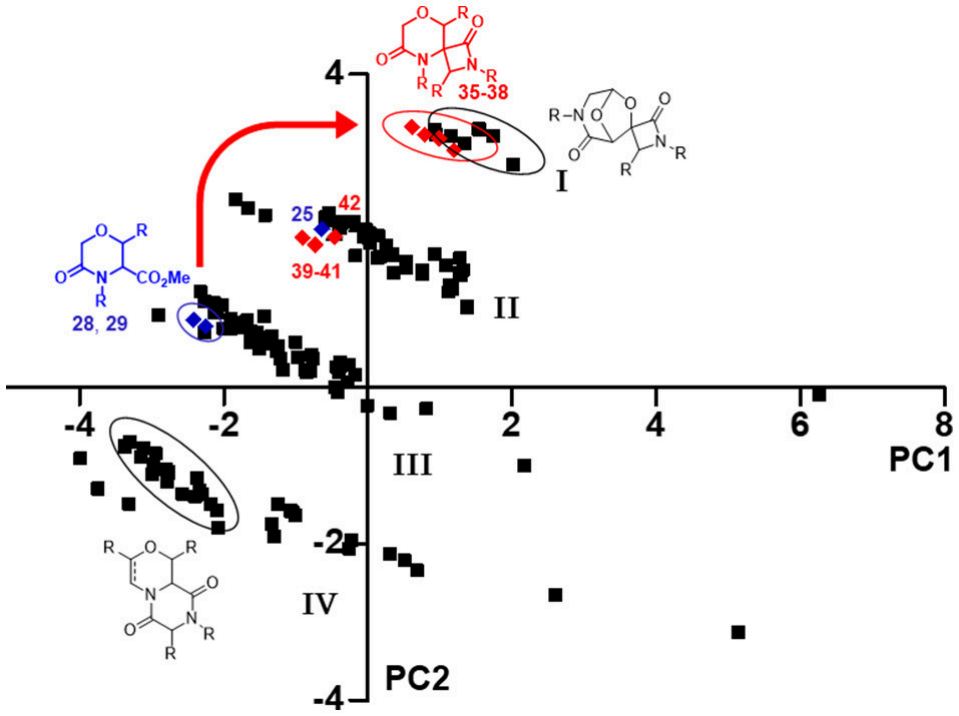
PCA plot resulting from the correlation between PC1 vs. PC2, showing the positioning in the chemical space of compounds **35**–**42** (red diamonds) and their parent analog **25**, **28**, and **29** (blue diamonds), in relation to an in-house library of 176 morpholine compounds (black squares). The thick red arrow indicates the shift in the chemical space induced by Staudinger chemistry from compounds **28–29** to spiro-β-lactams **35**–**38**. The ellipses highlight the various compounds subclasses; the compound clusters are numbered as **I**–**IV**.

Firstly, Principal Component Analysis (PCA), performed using the web-based public tool ChemGPS-NP, was used to simplify the comparison of all these molecules on the basis of different chemical properties (Xue et al., 2004; Tan, 2005). A pool of 186 compounds was analyzed, focusing in particular on principal component one (PC1), representing size, shape and polarizability, and the principal component two (PC2), that is a direct expression of aromatic and conjugation related properties, and plotted in a graph (Figure 5), where compounds **35**–**42** are shown as red diamonds, their parent analogs **25**, **28**, and **29** as blue diamonds, and the previously synthesized morpholines as black squares. All the library members were found being grouped in four different clusters (Figure 5, I–IV), depending on both the structure of the skeletons and side chain properties. As regarding to the introduction of quaternary stereocenters in the morpholine nucleus, a peculiar effect was found for the Staudinger reaction products. In fact, although the methylation did not induce any movement within the chemical space, as both compounds **25** and **42** reside in the second cluster, the Staudinger chemistry proved to shift the serine and threonine-derived morpholinone compounds **28–29** from the third cluster to the first one (Figure 5, red arrow), being populated also by spiro-β-lactams derived from the bicycle 3-aza-6,8-dioxabicyclo[3.2.1]octane, possibly due to the contribution to aromaticity given by the Staudinger reaction with aromatic imines.

**Figure 6:**
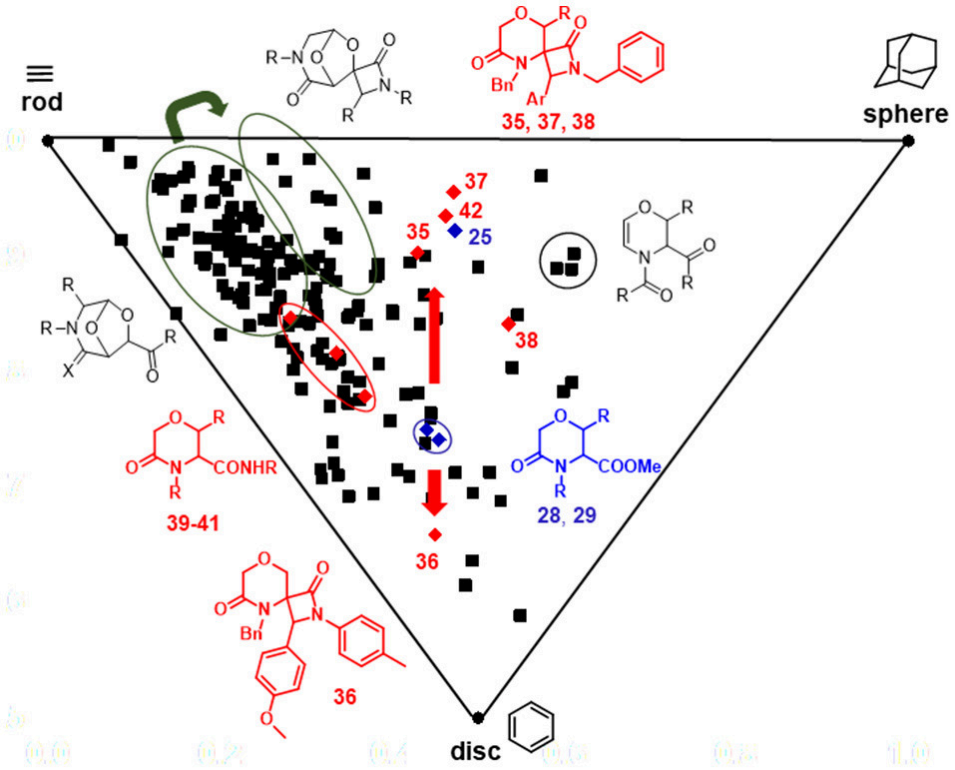
PMI plot showing the skeletal diversity of compounds **35–42** and their parent analogs **25**, **28**, and **29** (red diamonds), in relation to an in-house library of 176 morpholine compounds (black squares). The thick red arrows indicate the shift in the chemical space induced by Staudinger chemistry from compounds **28, 29** to spiro-β-lactams **35**–**38**, whereas the green arrow indicates the shift induced by the same chemistry performed on 6,8-dioxa-3-azabicyclo[3.2.1]octane core. The ellipses highlight the various compounds subclasses.

This significant movement in the chemical space achieved by the Staudinger chemistry was also observed in the Principal Moment of Inertia (PMI) analysis graph (Figure 6), obtained by calculating the three principal moments of inertia (I_xx_, I_yy_, I_zz_) and plotting their corresponding normalized values (I_1_/I_3_ and I_2_/I_3_) on a triangular graph, where the vertices (0,1), (0.5,0.5), and (1,1) represent a perfect rod (acetylene), disc (benzene) and sphere (adamantane), respectively (Sauer and Schwarz, 2003). As evinced from this graph, morpholine-derived compounds were found to lie along the center-left side of the triangle, as usually observed in the PMI analysis of small molecules. However, while the Staudinger chemistry performed on bicyclic 3-aza-6,8-dioxabicyclo[3.2.1]octanes did not result in a relevant shift in the PMI graph (Figure 6, green arrow), the installation of spiro-β-lactams on the morpholin-3-ones **28**–**29** proved to modulate significantly the three-dimensional complexity of these molecular frameworks. Compounds **28**–**29** were found to move from the center of the graph toward the rod-sphere axis (as for spiro-β-lactams **35**, **37, 38**) or the disc corner (as for spiro-β-lactam **36** that contains a *N*-*p*-tolyl group instead of a *N*-benzyl group) (Figure 6, red arrows). Also, amide by-products **39**–**41** were found lying closer to the rod-disc axis, as a result of the less three-dimensional character possessed by these structures, when compared to **28**–**29**. On the contrary, the effect of the extra methyl group in compound **42** did not prove to change significantly the shape of the morpholine nucleus, as this compound was found to be close to its parent **25** in the PMI plot. Interestingly, the bicyclic compounds based on the 6,8-dioxa-3-azabicyclo[3.2.1]octane core were found to be not close to the sphere region, as expected, possibly due to the major contribution in exploring the space toward the sphere-disc axis given by the side chains, as in the case of some dihydro-1,4-oxazine compounds with peculiar functional groups like the myristoyl chain.

**Figure 7:**
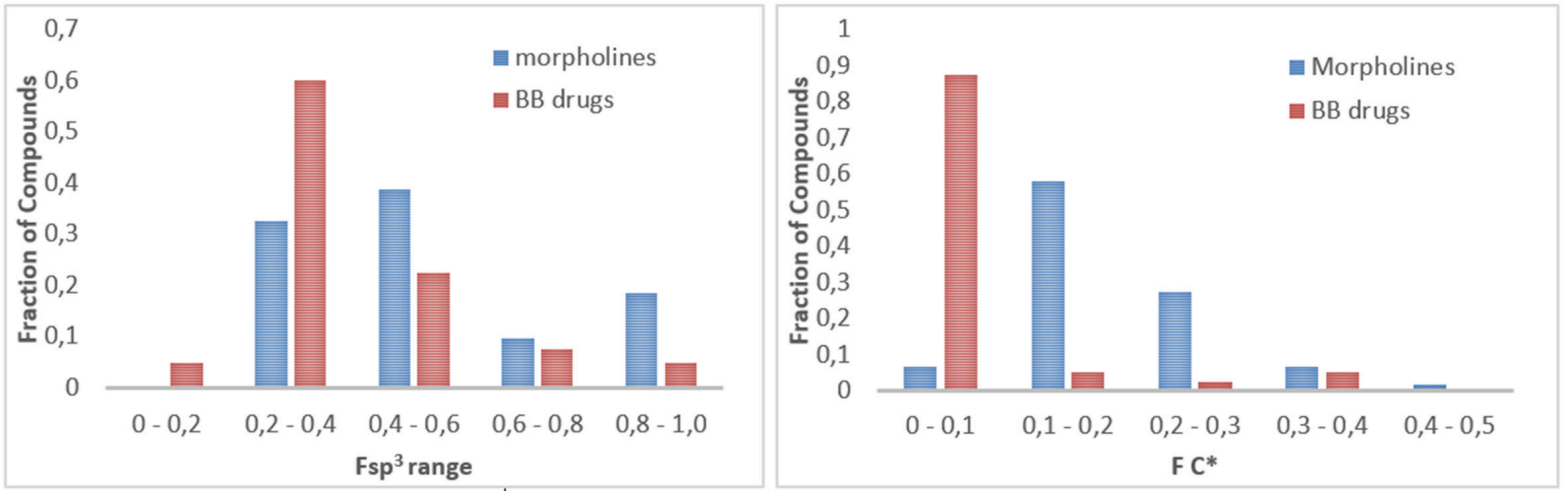
(**Left**) Fraction of compounds (morpholines in blue, blockbuster drugs in red) with different Fsp^3^ value, subdivided into 5 different ranges. (**Right**) Fraction of compounds (morpholines in blue, blockbuster drugs in red) with different FC^*^ value, subdivided into 5 different ranges.

To gain insight into a chemoinformatic evaluation of our in-house morpholine library, we calculated the saturation index (Fsp^3^) of each compound collection, as a measure of the molecular complexity (Lovering et al., 2009). This value was calculated as the ratio between the number of sp^3^ hybridized carbons in the molecule vs. the total carbon count and compared with those of a reference set of 40 brand-name blockbuster (BB) drugs as reported by Tan (Bauer et al., 2010; Kopp et al., 2012) (Figure 7, left). A similar approach was applied also to quantify the presence of stereocenters (Figure 7, right), by defining FC^*^ as the ratio of stereogenic center vs. the total carbon count. These two parameters (Fsp^3^ and FC^*^) allow to evaluate the quality of small molecule collections as regarding to the ability of both accessing new areas of the chemical space and giving successful results in drug discovery programmes. Sp^3^-rich DOS-derived small molecule collections proved to be more selective and more effective in binding to specific targets, as compared to analog small molecule libraries with lower Fsp^3^ ratio (Clemons et al., 2010), although the hit rate trend was found to be opposite in fragment-based screening (Hall et al., 2014). The analysis of the Fsp^3^ and FC^*^ parameters revealed that our library possesses higher frequency of molecules with a Fsp^3^ in the range between 0.4 and 0.6, as compared to the drugs, and also higher mean value of Fsp^3^ (Fsp^3^ morpholines = 0.52, Fsp^3^ BB drugs = 0.40) and FC^*^ ratio (FC^*^ morpholines = 0.19, FC^*^ BB drugs = 0.05). However, the Staudinger ketene-imine reaction, despite the possibility to introduce a quaternary stereocenter in the molecule, proved not to be a good strategy in terms of improving the Fsp^3^ ratio of the overall molecule, since it introduced a high number of sp^2^ carbon atoms due to the presence of aromatic appendages. In fact, the Fsp^3^ of starting compounds **28** and **29** (respectively 0.38 and 0.43) were reduced dramatically after the reaction to a mean value of 0.26 for the spiro-β-lactams **35**–**38**.

**Figure 8:**
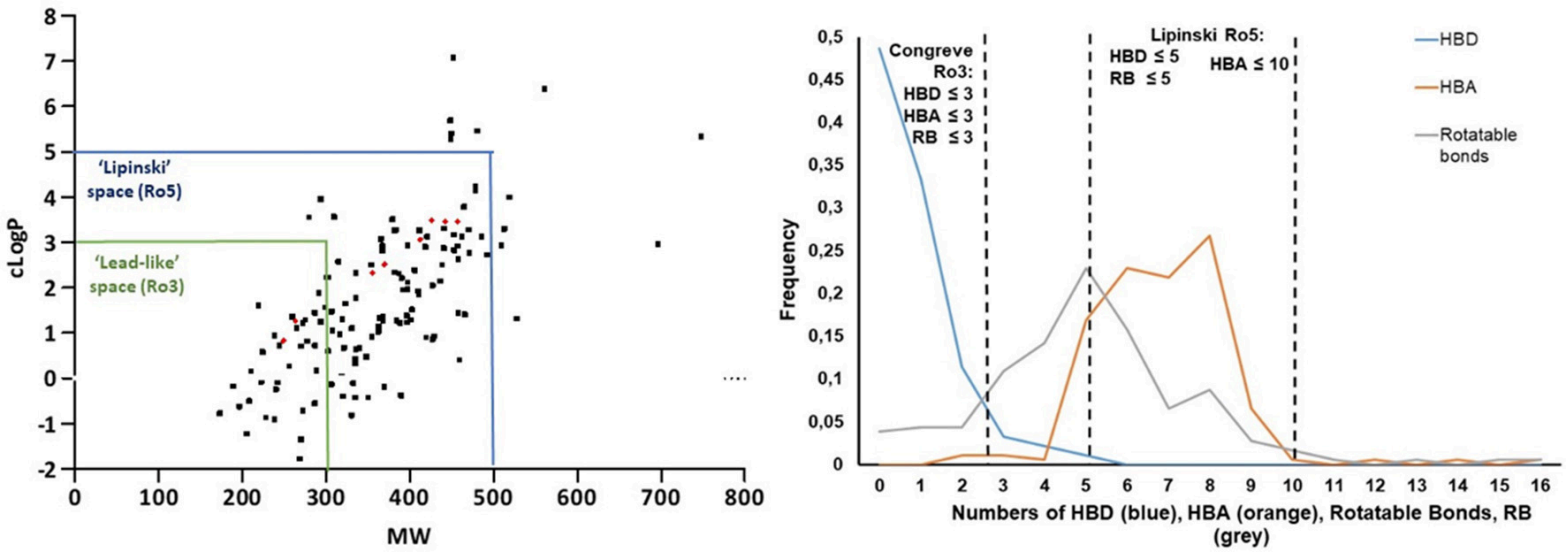
(**Left**) cLogP vs. molecular weight plot highlights all the compounds that follow Lipinski's “rule of five” (Lipiski space, blue box) and lead-likeness “rule of three” (Lead-like space, green box). (**Right**) Fraction of compounds with different number of hydrogen bond donors (HBD, blue line), hydrogen bond acceptors (HBA, orange line) and rotatable bonds (RB, gray line).

Finally, the investigation of small molecule physicochemical properties was carried out in order to establish the “druggability” and “lead-likeness” of our library, according to Lipinski's “rule of five” (Lipinski, 1997, 2004; Lipinski and Hopkins, 2004) and Congreve's “rule of three” (Congreve et al., 2003), respectively. In particular, we evaluated the lipophilicity and the molecular weight as key parameters to achieve good solubility, membrane permeability and subsequent oral bioavailability, by plotting clogP values (calculated as the logarithm of the partition coefficient between n-octanol and water) and the molecular weight of each library member in a graph (Figure 8, left). Only 12 out of 186 compounds were not compliant with Lipinski's “rule of five,” as they showed cLogP values higher than 5 and molecular weight higher than 500. This was evinced for compounds where morpholine was installed in a pentapeptide, or in the case of few bicyclic or morpholines characterized by a large number of aromatic substituents. Forty-five of these compounds were found following the restricted “lead-likeness” filters as proposed by the Congreve's “rule of three,” too, proving to be good starting points for potential drug optimization (Teague et al., 1999). Similarly, Veber et al. (2002) have proposed that the number of rotatable bonds (RB), together with the number of hydrogen bond donors (HBD) and hydrogen bond acceptors (HBA), can give another good criteria for predicting oral bioavailability. According to such structural parameters, only 8 compounds of our library were found not following the Veber's rule (RB ≤ 10 and (HBA + HBD) ≤ 12) for a good bioavailability. The graph reported in Figure 8, right can easily show that most of the morpholine compounds are within the cut-off values of drug-like Lipinski's “rule of five” (HBA ≤ 10, HBD ≤ 5, RB ≤ 5), whereas only for the number of HBD (blue line) the Congreve lead-like “rule of three” is satisfied (HBD ≤ 3, HBA ≤ 3, RB ≤ 3) (Figure 8, right). As expected, no particular changing in the Lipinski drug-like properties were observed for the spiro-β-lactams **35**–**38** derived from the Staudinger reaction, since the molecular weight and the cLogP values increased significantly, but still remained under the cut-off values of Lipinski's “rule of five,” as well as the number of HBA, HBD and rotatable bonds. In particular, the introduction of the nitrogen atom brought another hydrogen bond acceptor to the molecule (moving from 5 to 6) and the number of rotatable bonds increased from 4 to 5 or 6, depending on the imine counterpart.

## Conclusions

The development of new peptidomimetic scaffolds useful to address protein-protein interactions is still a growing field of medicinal chemistry and chemical biology. This approach requires efficient synthetic processes able to produce high-quality small molecule collections, as in the case of the use of Diversity Oriented Synthesis (DOS) strategies, especially starting from amino acid and sugar derivatives, to produce polyfunctional and sp^3^-rich building blocks. Our efforts in this field are focused on the generation of different peptidomimetic compounds around the morpholine nucleus, as this heterocycle is contained in many different bioactive molecules.

In order to increase the complexity and the sp^3^ character of this important nucleus, we studied different build/couple/pair strategies that exploit complexity-generating reactions. In this work, as a further improvement in this direction, we envisioned to transform the sp^3^ carbon atom in α-position of the carbomethoxy group of selected morpholin-3-one starting materials, by means of the Staudinger reaction, to generate morpholinone-derived spiro-β-lactams and of different alkylation strategies. This approach proved to be valuable, especially when assessing the structural diversity and complexity of these new compounds in comparison with 176 morpholine-derived small molecules previously synthesized in our laboratories, by analyzing the populated chemical space. In fact, both PCA (Principal Component Analysis) and PMI (Principal Moment of Inertia) analysis revealed that the Staudinger ketene-imine reaction proved to shift the serine and threonine-derived morpholine-3-one compounds in new areas of the chemical space, assessing a relevant change of positions, hardly achieved by using other synthetic approaches. Finally, we also investigated different small-molecule physicochemical parameters (cLogP, molecular weight, number of rotatable bonds, hydrogen bond acceptors, hydrogen bond donators, Fsp^3^, FC^*^) of all the 186 morpholines of the library in comparison with a reference set of 40 brand-name blockbuster (BB) drugs. These analyses revealed that only few compounds did not show “drug-like” values, as defined by the Lipinski rule of five, whereas most of the compounds showed higher Fsp^3^ and FC^*^ values as compared to the drugs. Indeed, several applications in medicinal chemistry projects demonstrated over the years the value of morpholine as a scaffold for peptidomimetic design and drug discovery.

## Author contributions

AT and EL conceived the research. EL and RI carried out the synthesis. EL carried out the chemoinformatics analyses. AT carried out the molecular modeling calculations. AT and GM supervised the work. EL and AT wrote the paper. All the authors revised the manuscript.

### Conflict of interest statement

The authors declare that the research was conducted in the absence of any commercial or financial relationships that could be construed as a potential conflict of interest.
