# Supplementary material for: Diversity-Oriented Synthesis and Chemoinformatic Analysis of the Molecular Diversity of sp3-Rich Morpholine Peptidomimetics
Source: Front Chem. 2018 Oct 30;6:522. doi: 10.3389/fchem.2018.00522 (PMC6218424; doi:10.3389/fchem.2018.00522)
Supplement: Supplementary file 1 [file Table_1.DOCX]

Diversity-Oriented Synthesis and chemoinformatic analysis of the molecular diversity of sp^3^-rich morpholine peptidomimetics

Elena Lenci,^*^ Riccardo Innocenti, Gloria Menchi, Andrea Trabocchi^[[1]](#footnote-1)^*

Department of Chemistry “Ugo Schiff”, University of Florence, Via della Lastruccia 13, 50019, Sesto Fiorentino, Florence, Italy

SUPPLEMENTARY MATERIAL

Experimental procedures and characterization data for **25**, **28**-**31**, **35**-**38**, **42** S2-S6

^1^H and ^13^C NMR spectra for compounds **29**, **35**-**38**, **42** S7-S12

NOESY1D spectra of compounds **37** and **42** S13-S14

PCA data for morpholine library compounds S15-S16

PMI data for morpholine library compounds S17-S20

Medicinally-relevant molecular properties (MW, ClogP, HBD, HBA,

rotatable bonds count, Fsp^3^ and FC*) for morpholine library compounds S21-S25

Fsp^3^ and FC* data for a reference set of BB drugs S26

References S27

**General**

^1^H NMR and ^13^C NMR spectra were recorded on a Varian Mercury 400 (^1^H: 400 MHz, ^13^C: 100 MHz), or a Varian Gemini 200 (^1^H: 200 MHz, ^13^C: 50 MHz). The chemical shifts (*δ*) and coupling constants (*J*) are expressed in parts per million (ppm) and hertz (Hz), respectively. Flash column chromatography (FCC) purifications were performed manually using glass columns with Merck silica gel (0.040–0.063 mm), or using the Biotage Isolera system and SNAP silica cartridges. TLC analyses were performed on Merck silica gel 60 F254 plates. Melting points were recorded on a Büchi B-540 instrument and are uncorrected. Elemental analyses were performed on a Perkin Elmer 240 C, H, N analyzer. ESI mass spectra were recorded on a Thermo LCQ-Fleet. Microwave heated reactions were performed using a Biotage Initiator instrument. Optical rotation measurements were performed on a JASCO DIP-370 polarimeter and are given in 10^−1^ deg cm^2^ g^−1^. All commercially available reagents and solvents were used as received, unless otherwise specified.

*trans*-Methyl 4-benzyl-3-(4-methoxyphenyl)-5-oxomorpholine-2-carboxylate (**25**)

A mixture of 1,4-dioxane-2,6-dione (340 mg, 2.93 mmol) and *N*-(4-methoxybenzylidene)-1-phenylmethanamine (660 mg, 2.93 mmol) in toluene (13 mL) was heated at 80 °C and stirred for 4 hours at this temperature, as reported in literature. Then, the crude mixture was dissolved in MeOH (29.3 mL), SOCl_2_ (1.1 mL, 5.86 mmol) was added dropwise and the mixture was left refluxing for 2 h. After solvent evaporation, the crude mixture was purified by flash chromatography (EtOAc/Petr. et. = 1:1) to give compound **25** (696 mg, 1.96 mmol) in 67% overall yield. Spectroscopical data are in agreement with values reported in the literature.^[[2]](#endnote-1)^

(*S*)-Methyl 4-benzyl-5-oxomorpholine-3-carboxylate (**28**)

To a solution of bromoacetyl bromide (200 μL, 2.29 mmol) and Et_3_N (478 μL, 3.44 mmol) in dry CH_2_Cl_2_ (23 mL), a solution of *N*-benzylserine (446 mg, 2.29 mmol) in dry CH_2_Cl_2_ (23 mL) was added slowly at −15 °C. The resulting mixture was left stirring at the same temperature for 1 h, then the solvent was evaporated under reduced pressure. After dissolving the crude mixture (450 mg, 1.37 mmol) in anhydrous THF (27 mL), sodium hydride (60% in oil, 110 mg, 2.74 mmol) was added slowly at 0 °C, and the mixture was left stirring for 1 h at room temperature. NaH was quenched with MeOH (10 mL) and filtered through Celite. The filtrate was concentrated under vacuum, to yield a yellow oil that was purified by flash chromatography (EtOAc/Petr. et. = 1:1) affording pure compound **28** (367 mg, 1.47 mmol) in 64% overall yield. Spectroscopical data are in agreement with values reported in the literature.^[[3]](#endnote-2)^

(2*R*,3*S*)-Methyl 4-benzyl-2-methyl-5-oxomorpholine-3-carboxylate (**29**)

To a solution of bromoacetyl bromide (260 μL, 3.00 mmol) and Et_3_N (630 μL, 4.50 mmol) in dry CH_2_Cl_2_ (30 mL), a solution of *N*-benzylthreonine (630 mg, 3.00 mmol) in dry CH_2_Cl_2_ (30 mL) was added slowly at −15 °C. The resulting mixture was left stirring at the same temperature for 1 h, then the solvent was evaporated under reduced pressure. After dissolving the crude mixture (541 mg, 1.57 mmol) in anhydrous THF (30 mL), sodium hydride (60% in oil, 125 mg, 3.15 mmol) was added slowly at 0 °C, and the mixture was left stirring for 1 h at room temperature. NaH was quenched with MeOH (10 mL) and filtered through Celite. The filtrate was concentrated under vacuum, to yield a yellow oil that was purified by flash chromatography (EtOAc/Petr. et. = 1:2) affording pure compound **29** (386 mg, 1.47 mmol) in 55% overall yield. [α]_D_^21^ = + 46.7 (CHCl_3_, *c* = 1.2). ^1^H NMR (400 MHz, CDCl_3_) δ 7.33 – 7.22 (m, 5H, CH_ar_ x 5), 5.47 (d, *J* = 14.8 Hz, 1H, CH_2a_-Ph), 4.27 (m, 2H, CH_2_-6), 4.20 (q, *J* = 4.6 Hz, 1H, CH-2), 3.79 (d, *J* = 14.8 Hz, 1H, CH_2b_-Ph), 3.72 (s, 3H, OCH_3_), 3.71 (s, 1H, CH-3), 1.23 (d, *J* = 6.5 Hz, 3H, CH_3_).^13^C NMR (100 MHz, CDCl_3_) δ 169.9 (CO_2_), 166.8 (CO), 135.3 (C_ar_), 128.8 (2C, CH_ar_ x 2), 128.7 (2C, CH_ar_ x 2), 127.9 (CH_ar_), 70.6 (CH-2), 64.6 (CH-3), 62.7 (CH_2_-6), 52.7 (OCH_3_), 48.3 (CH_2_-Ph), 16.6 (CH_3_). MS (ESI) m/z (%): 286.32 [(M + Na)^+^, 100]. Anal. Calcd. for C_14_H_17_NO_4_: C, 63.87; H, 6.51; N, 5.32. Found: C, 63.98; H, 6.64; N, 5.17.

(*S*)-4-Benzyl-5-oxomorpholine-3-carbonyl chloride (**30**)

Morpholine-3-one **28** (367 mg, 1.47 mmol) was dissolved in THF (9 mL), then 1 M aqueous solution of LiOH (3 mL, 3 mmol) was added slowly at 0 °C. After stirring for 16 h at room temperature, the mixture was diluted with EtOAc and acidified to pH 1 with a 1 M solution of HCl. Then, the aqueous phase was extracted three times with EtOAc, dried with Na_2_SO_4_ and concentrated under vacuum. The resulting pure acid (258 mg, 1.10 mmol) was then dissolved in SOCl_2_ (810 µL, 11.00 mmol) and left refluxing for 2 h under a nitrogen atmosphere. After removing the volatiles under reduced pressure, acyl chloride **30** (290 mg, 1.15 mmol, 78% overall yield) was obtained and immediately used in the Staudinger reaction.

(2*R*,3*S*)-4-Benzyl-2-methyl-5-oxomorpholine-3-carbonyl chloride (**31**)

Morpholine-3-one **29** (305 mg, 1.15 mmol) was dissolved in THF (7 mL), then 1 M aqueous solution of LiOH in water (2.3 mL, 2.3 mmol) was added slowly at 0 °C. After stirring for 16 h at room temperature, the mixture was diluted with EtOAc and acidified to pH 1 with a 1 M solution of HCl. Then, the aqueous phase was extracted three times with EtOAc, dried with Na_2_SO_4_ and concentrated under vacuum. The resulting pure acid (300 mg, 1.15 mmol) was then dissolved in SOCl_2_ (838 µL, 11.50 mmol) and left refluxing for 2 h under a nitrogen atmosphere. After removing the volatiles under reduced pressure, acyl chloride **31** (258 mg, 0.96 mmol, 84% overall yield) was obtained and immediately used in the Staudinger reaction.

General procedure for the synthesis of imines.

A solution of the corresponding amine (1 eq.) and aldehyde (1 eq.) in dichloromethane (2 mL / mmol) was kept over MgSO_4_ (1.5 eq.) at room temperature for 16 hours. After completion of the reaction, the insoluble material was filtered off and the filtrate was concentrated under reduced pressure, giving the corresponding pure imine that was immediately used in the Staudinger reaction.

General procedure for the synthesis of spiro compounds.

A solution of the proper imine (1.1 eq.) and dry TEA (1.5 eq.) in dry toluene (5 mL / mmol) was heated to reflux under a nitrogen atmosphere; then a mixture of morpholine-3-one acyl chloride (1 eq.) in dry toluene (2.5 mL / mmol) was added. The mixture was stirred at 110 °C under a nitrogen atmosphere for 16 hours. Then, the organic phase was washed with a saturated NaHCO_3_ aqueous solution and brine. The organic phase was dried with Na_2_SO_4_, filtered and concentrated under reduced pressure to give a dark oil that was purified by flash chromatography.

(±)-(3*S*,4*S*)-2,5-Dibenzyl-3-phenyl-8-oxa-2,5-diazaspiro[3.5]nonane-1,6-dione (**35**)

Compound **35** was obtained following the general Staudinger procedure starting from serine-derived morpholine-3-one acyl chloride **30** (66 mg, 0.26 mmol) and *N*-benzylidene-1-phenylmethanamine (51 mg, 0.26 mmol). The crude product was purified by flash chromatography (EtOAc/Petr. et. = 1:2) affording compound **35** (56 mg, 52%) as a racemic mixture of the (3,4)-*cis*-stereoisomer. ^1^H NMR (400 MHz, CDCl_3_) δ 7.48 – 7.30 (m, 7H, CH_ar_ x 7), 7.30 – 7.13 (m, 8H, CH_ar_ x 8), 5.11 (d, *J* = 14.6 Hz, 1H, *N*(5)-CH_2a_-Ph), 4.76 (s, 1H, CH-3), 4.20 (m, 3H, *N*(5)-CH_2b_-Ph + CH_2_-7), 4.09 (d, *J* = 11.8 Hz, 1H, CH_2a_-9), 3.94 (dd, *J* = 13.4, 8.9 Hz, 2H, CH_2b_-9 + *N*(2)-CH_2a_-Ph), 3.69 (d, *J* = 15.1 Hz, 1H, *N*(2)-CH_2b_-Ph).^13^C NMR (100 MHz, CDCl_3_) δ 166.4 (CO), 164.4 (CO), 136.5 (C_ar_), 134.5 (C_ar_), 134.2 (C_ar_), 129.2 (4C, CH_ar_ x 4), 128.7 (2C, CH_ar_ x 2), 128.5 (2C, CH_ar_ x 2), 128.1 (2C, CH_ar_ x 2), 127.7 (2C, CH_ar_ x 2), 127.1 (CH_ar_), 126.2 (2C, CH_ar_ x 2), 78.2 (C-4), 69.4 (CH_2_-9), 68.2 (CH_2_-7), 66.3 (CH-3), 50.6 (*N*(2)-CH_2_-Ph), 46.2 (*N*(5)-CH_2_-Ph). MS (ESI) m/z (%): 435.29 [(M + Na)^+^, 100]. Anal. Calcd. for C_26_H_24_N_2_O_3_: C, 75.71; H, 5.86; N, 6.79. Found: C, 75.99; H, 5.98; N, 6.65.

(±)-(3*SR*,4*S*)-5-Benzyl-3-(4-methoxyphenyl)-2-(*p*-tolyl)-8-oxa-2,5-diazaspiro[3.5]nonane-1,6-dione (**36**)

Compound **36** was obtained following the general Staudinger procedure starting from serine-derived morpholine-3-one acyl chloride **30** (223 mg, 0.87 mmol) and *N*-(4-methoxybenzylidene)-4-methylaniline (196 mg, 0.87 mmol). The crude product was purified by flash chromatography (EtOAc/Petr. et. = 1:3) affording compound **36** (116 mg, 35%) as a 3:1 mixture of diastereoisomer in favour of the *cis*-isomer. ^1^H NMR (400 MHz, CDCl_3_) major diastereoisomer δ 7.34 – 6.79 (m, 26H, CH_ar_ x 26, Major and minor), 5.66 (d, *J* = 15.6 Hz, 1H, *N*(5)-CH_2a_-Ph, Major), 5.41 (s, 1H, CH-3, minor), 4.74 (s, 1H, CH-3, Major), 4.40 – 4.16 (m, 6H, CH_2_-7, Major and minor, + CH_2_-9, minor), 4.13 – 4.03 (m, 1H, *N*(5)-CH_2b_-Ph, Major), 3.94 (d, *J* = 15.2 Hz, 1H, *N*(5)-CH_2a_-Ph, minor), 3.87 – 3.74 (m, 3H, *N*(5)-CH_2b_-Ph, minor, + CH_2_-9, major), 3.82 (s, 3H, OCH_3_, minor), 3.75 (s, 3H, OCH_3_, Major), 2.33 (s, 3H, CH_3_, minor), 2.30 (s, 3H, CH_3_, Major). ^13^C NMR (100 MHz, CDCl_3_) δ 167.4 (CO, Major), 166.6 (CO, minor), 162.3 (CO, Major), 161.1 (CO, minor), 159.8 (C_ar_-OMe, Major), 159.7 (C_ar_-OMe, minor), 137.5 (C_ar_, Major), 136.6 (C_ar_, minor), 134.9 (C_ar_, minor), 134.8 (C_ar_, minor), 134.7 (C_ar_, Major), 133.5 (C_ar,_ Major), 129.8 (2C, CH_ar_ x 2, Minor), 129.7 (2C, CH_ar_ x 2, minor), 128.9 (2C, CH_ar_ x 2, Major), 128.6 (2C, CH_ar_ x 2, Major), 128.1 (CH_ar,_ Major), 128.0 (2C, CH_ar_ x 2, Major), 127.9 (CH_ar_, minor), 127.8 (2C, 2 x CH_ar_, minor), 127.6 (2C, CH_ar_ x 2, Major), 127.2 (C_ar_, minor), 127.1 (C_ar_, Major) 124.9 (CH_ar_, minor), 123.9 (CH_ar_, minor), 118.0 (2C, CH_ar_ x 2, Major), 117.5 (2C, CH_ar_ x 2, minor), 114.6 (2C, CH_ar_ x 2, minor), 114.3 (2C, CH_ar_ x 2, Major), 75.8 (2C, C-4, Major and minor), 70.2 (CH_2_-9, minor), 68.3 (CH_2_-7, minor), 68.2 (CH_2_-9, Major), 66.6 (CH-3, minor), 65.3 (CH_2_-7, Major), 63.9 (CH-3, Major), 55.3 (OCH_3_, minor), 55.2 (OCH_3_, Major), 50.8 (*N*(5)-CH_2_-Ph, minor), 46.6 (*N*(5)-CH_2_-Ph, Major), 21.0 (2C, CH_3_, Major and minor). MS (ESI) m/z (%): 465.41 [(M + Na)^+^, 100]. Anal. Calcd. for C_27_H_26_N_2_O_4_: C, 73.28; H, 5.92; N, 6.33. Found: C, 73.75; H, 6.05; N, 6.17.

(3*S*,4*S*,9*R*)-2,5-Dibenzyl-9-methyl-3-phenyl-8-oxa-2,5-diazaspiro[3.5]nonane-1,6-dione (**37**)

Compound **37** was obtained following the general Staudinger procedure starting from threonine-derived morhpoline-3-one acyl chloride **31** (130 mg, 0.48 mmol) and *N*-benzylidene-1-phenylmethanamine (93 mg, 0.48 mmol). The crude product was purified by flash chromatography (EtOAc/Petr. et. = 1:2) affording compound **37** (10 mg, 15%) as a single stereoisomer together with small impurities of the amide by-product. [α]_D_^20^ = + 59.3 (CHCl_3_, *c* = 0.8). ^1^H NMR (400 MHz, CDCl_3_) δ 7.51 – 7.11 (m, 15H, CH_ar_ x 5), 5.03 (d, *J* = 14.3 Hz, 1H, *N*(5)-CH_2a_-Ph), 4.84 (s, 1H, CH-3), 4.26 – 4.08 (m, 4H, *N*(5)-CH_2b_-Ph + CH_2_-7 + CH-9), 4.02 – 3.94 (d, *J* = 15.1 Hz, 1H, *N*(2)-CH_2a_-Ph), 3.78 (d, *J* = 15.1 Hz, 1H, *N*(2)-CH_2b_-Ph), 1.00 (d, *J* = 6.5 Hz, 3H, CH_3_).^13^C NMR (50 MHz, CDCl_3_) δ 166.7 (CO), 164.5 (CO), 136.9 (C_ar_), 134.5 (C_ar_), 134.4 (C_ar_), 129.5 (2C, CH_ar_ x 2), 129.2 (2C, CH_ar_ x 2), 129.0 (2C, CH_ar_ x 2), 128.6 (CH_ar_), 128.5 (CH_ar_), 128.1 (2C, CH_ar_ x 2), 127.8 (2C, CH_ar_ x 2), 127.1 (CH_ar_), 126.5 (2C, CH_ar_ x 2), 81.2 (C-4), 74.2 (CH-9), 68.4 (CH_2_-7), 61.7 (CH-3), 51.2 (*N*(2)-CH_2_-Ph), 46.4 (*N*(5)-CH_2_-Ph), 15.5 (CH_3_). MS (ESI) m/z (%): 449.49 [(M + Na)^+^, 100]. Anal. Calcd. for C_27_H_26_N_2_O_3_: C, 76.03; H, 6.14; N, 6.57. Found: C, 76.34; H, 6.26; N, 6.24.

(3*S*,4*S*,9*R*)-2,5-Dibenzyl-3-(4-methoxyphenyl)-9-methyl-8-oxa-2,5-diazaspiro[3.5]nonane-1,6-dione (**38**)

Compound **38** was obtained following the general Staudinger procedure starting from threonine-derived morhpoline-3-one acyl chloride **31** (80 mg, 0.30 mmol) and *N*-(4-methoxybenzylidene)-1-phenylmethanamine (68 mg, 0.30 mmol). The crude product was purified by flash chromatography (EtOAc/Petr. et. = 1:3) affording compound **38** (26 mg, 19%) as a single stereoisomer together with small impurities of the amide by-product. [α]_D_^23^ = + 18.9 (CHCl_3_, *c* = 0.7). ^1^H NMR (400 MHz, CDCl_3_) δ 7.46 – 7.10 (m, 12H, CH_ar_ x 12), 6.99 – 6.86 (m, 2H, CH_ar_ x 2), 5.00 (d, *J* = 14.2 Hz, 1H, *N*(5)-CH_2a_-Ph), 4.77 (s, 1H, CH-3), 4.21 – 4.13 (m, 4H, *N*(5)-CH_2b_-Ph + CH_2_-7 + CH-9), 4.11 – 4.03 (m, 1H, *N*(2)-CH_2a_-Ph), 3.82 (s, 3H, OCH_3_), 3.82 – 3.76 (m, 1H, *N*(2)-CH_2b_-Ph), 1.00 (d, *J* = 6.5 Hz, 3H, CH_3_). ^13^C NMR (50 MHz, CDCl_3_) δ 166.7 (CO), 163.1 (CO), 159.8 (C_ar_-OMe), 137.0 (C_ar_), 134.3 (C_ar_), 129.5 (2C, CH_ar_ x 2), 129.0 (2C, CH_ar_ x 2), 128.5 (CH_ar_), 128.1 (2C, CH_ar_ x 2), 127.8 (2C, CH_ar_ x 2), 127.7 (2C, CH_ar_ x 2), 127.1 (CH_ar_), 121.9 (C_ar_), 114.7 (2C, CH_ar_ x 2), 79.8 (C-4), 74.0 (CH-9), 68.3 (CH_2_-7), 61.3 (CH-3), 55.3 (OCH_3_), 51.3 (*N*(5)-CH_2_-Ph), 46.2 (*N*(2)-CH_2_-Ph), 15.5 (CH_3_). MS (ESI) m/z (%): 479.33 [(M + Na)^+^, 100]. Anal. Calcd. for C_28_H_28_N_2_O_4_: C, 73.66; H, 6.18; N, 6.14. Found: C, 73.89; H, 6.45; N, 5.98.

(±)-(2*S*,3*S*)-methyl 4-benzyl-3-(4-methoxyphenyl)-2-methyl-5-oxomorpholine-2-carboxylate (**42**)

A solution of **25** (645 mg, 1.96 mmol) in anhydrous THF (13 mL) was cooled to -78 °C, then a 1 M NaHMDS solution in THF (2.94 mL, 2.94 mmol) was added dropwise. The resulting solution was left stirring at -78 °C under a nitrogen atmosphere for 1 h, then methyl iodide (134 µL, 2.16 mmol) was slowly added. The reaction mixture was allowed to return to room temperature and left stirring for 16 hours at such temperature. Successively, the reaction mixture was quenched with a 1:1 mixture of EtOAc (50 mL) and 1 M HCl (50 mL), the aqueous phase was discarded and the organic phase was washed with 1 M HCl (3x 30 mL), brine (30 mL) and dried over sodium sulfate. After solvent evaporation, the crude product was purified by flash chromatography (EtOAc/Hexane = 1:1) to give pure compound **42** in 72% yield. ^1^H NMR (400 MHz, CDCl_3_) δ 7.39 – 7.28 (m, 3H, CH_ar_ x 3), 7.23 (dd, *J* = 8.6, 7.1 Hz, 2H, CH_ar_ x 2), 7.13 (d, *J* = 8.7 Hz, 2H, CH_ar_ x 2), 6.86 (d, *J* = 8.6 Hz, 2H, CH_ar_ x 2), 5.54 (d, *J* = 14.5 Hz, 1H, CH_2a_-Ph), 4.65 (d, *J* = 17.5 Hz, 1H, CH_2b_-6), 4.44 (d, *J* = 17.5 Hz, 1H, CH_2a_-6), 4.13 (s, 1H, CH-2), 3.79 (s, 3H, O-CH_3_), 3.38 (s, 3H, CO_2_CH_3_), 3.26 (d, *J* = 14.5 Hz, 1H, CH_2b_-Ph), 1.53 (s, 3H, CH_3_).^13^C NMR (100 MHz, CDCl_3_) δ 170.5 (CO_2_), 165.5 (C-5), 159.9 (C_ar_), 135.8 (C_ar_), 129.7 (2C, CH_ar_ x 2), 128.9 (2C, CH_ar_ x 2), 128.8 (2C, CH_ar_ x 2), 128.0 (CH_ar_), 127.2 (C_ar_), 113.9 (2C, CH_ar_ x 2), 78.3 (C-2), 63.1 (CH_2_-6), 62.8 (CH-3), 55.3 (OCH_3_), 52.3 (CO_2_CH_3_), 46.8 (CH_2_-Ph), 19.5 (CCH_3_). MS (ESI) m/z (%): 392.44 [(M + Na)^+^, 100]. Anal. Calcd. for C_21_H_23_NO_5_: C, 68.28; H, 6.28; N, 3.79. Found: C, 68.55; H, 6.41; N, 3.51.


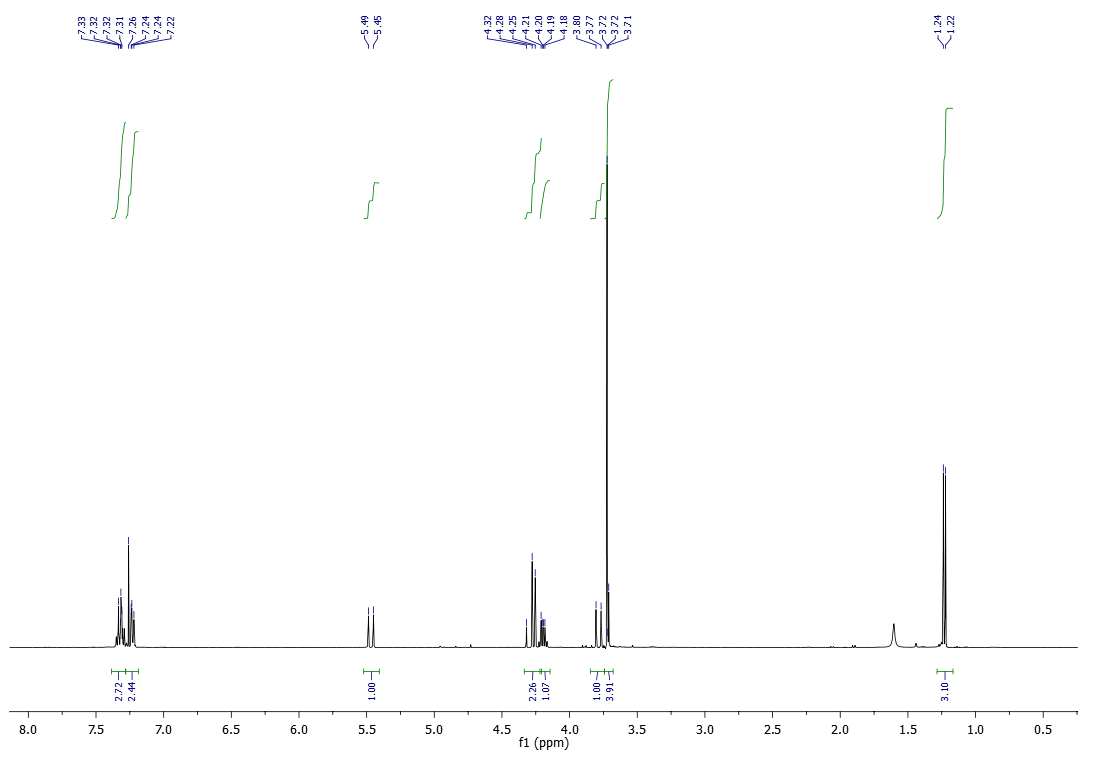


Figure S1. ^1^H NMR spectrum of compound **29** (400 MHz, CDCl_3_).


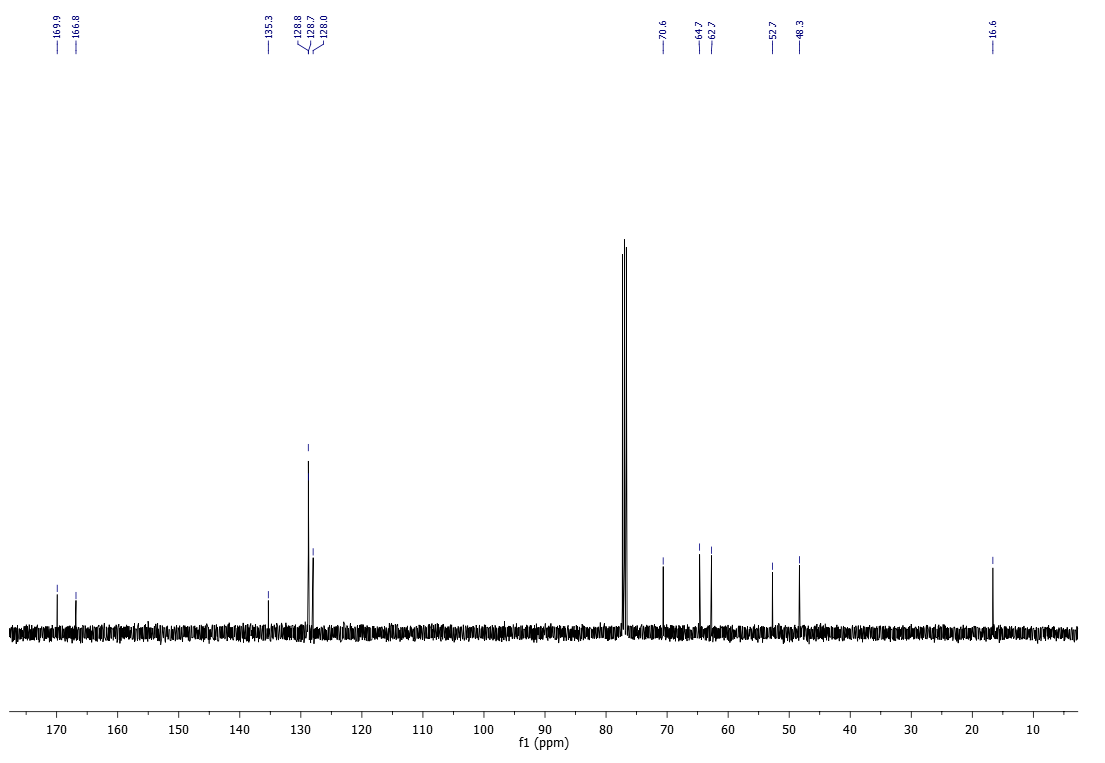


Figure S2. ^13^C NMR spectrum of compound **29** (100 MHz, CDCl_3_).


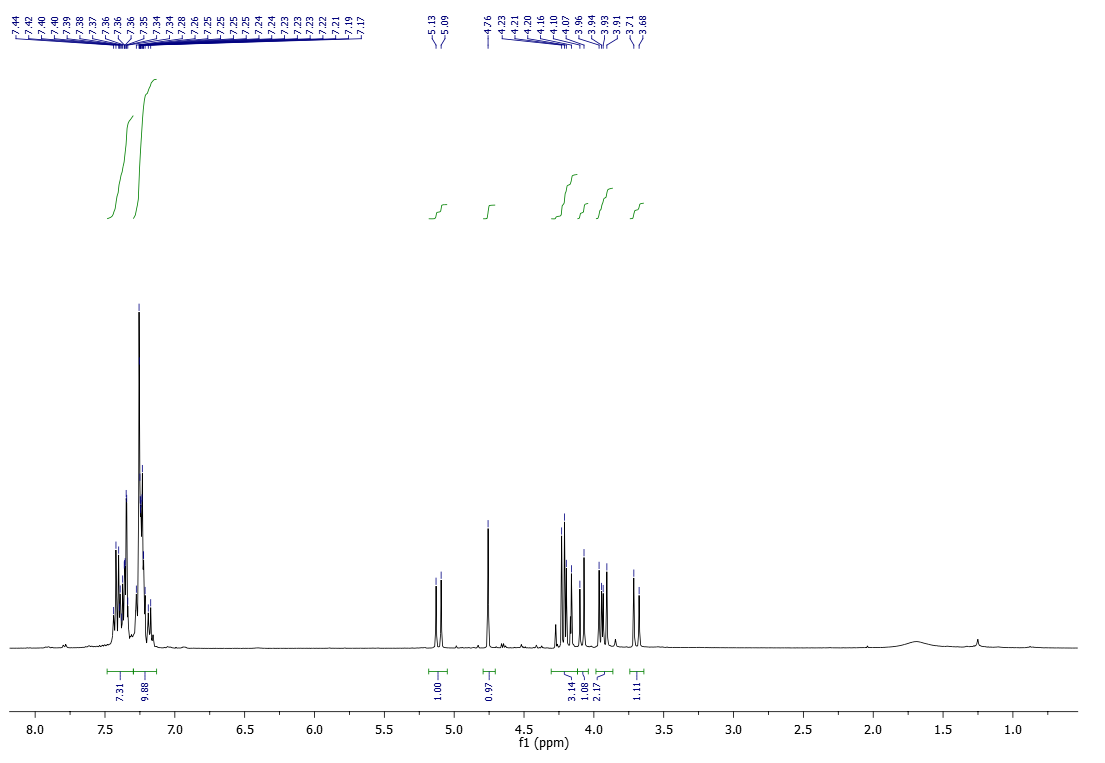
Figure S3. ^1^H NMR spectrum of compound **35** (50 MHz, CDCl_3_).

**
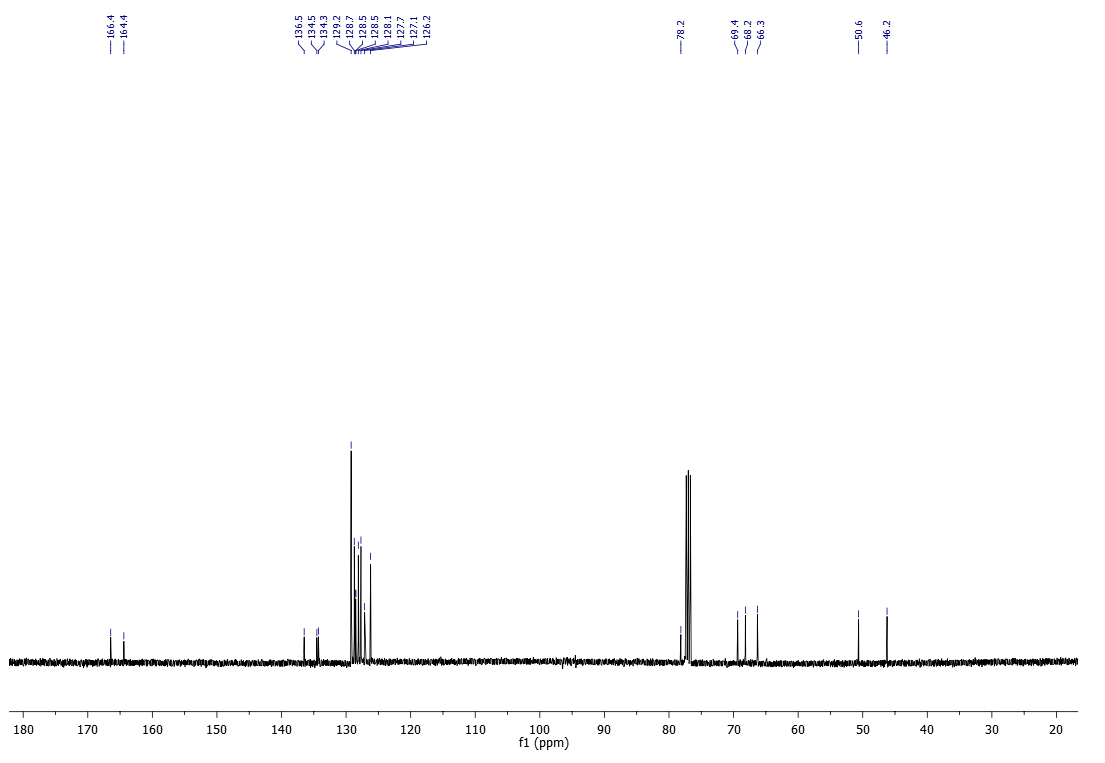
**

Figure S4. ^13^C NMR spectrum of compound **35** (100 MHz, CDCl_3_).


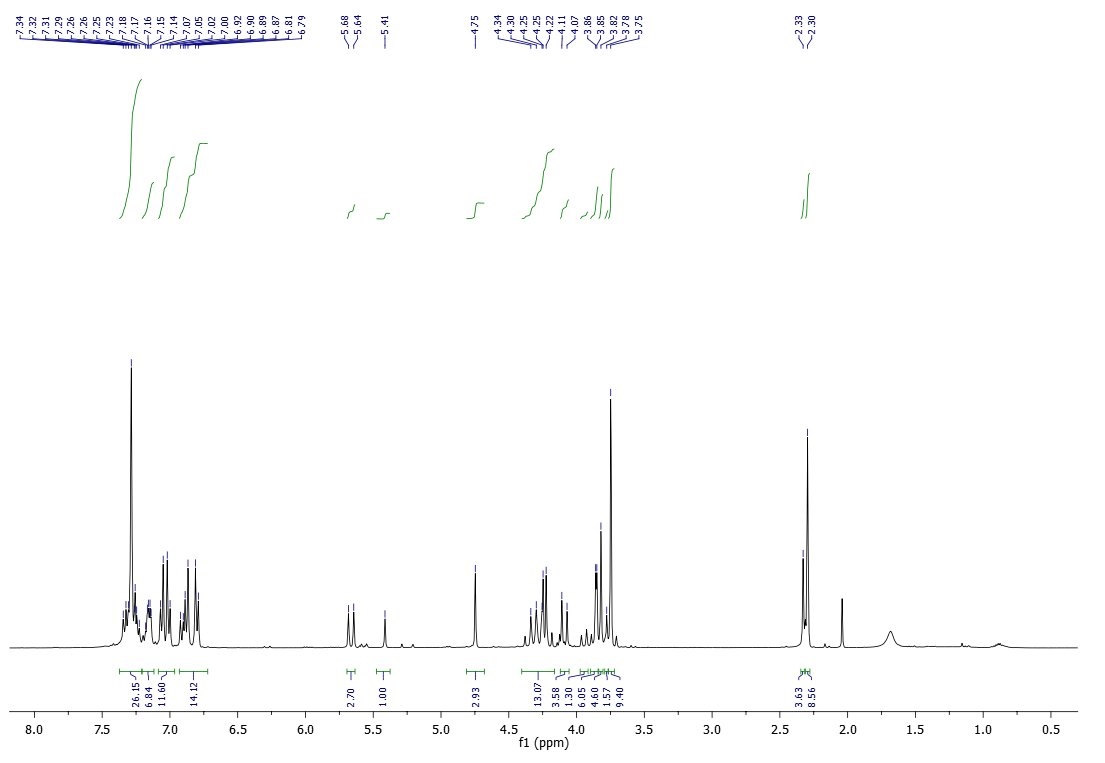


Figure S5. ^1^H NMR spectrum of compound **36** (400 MHz, CDCl_3_).


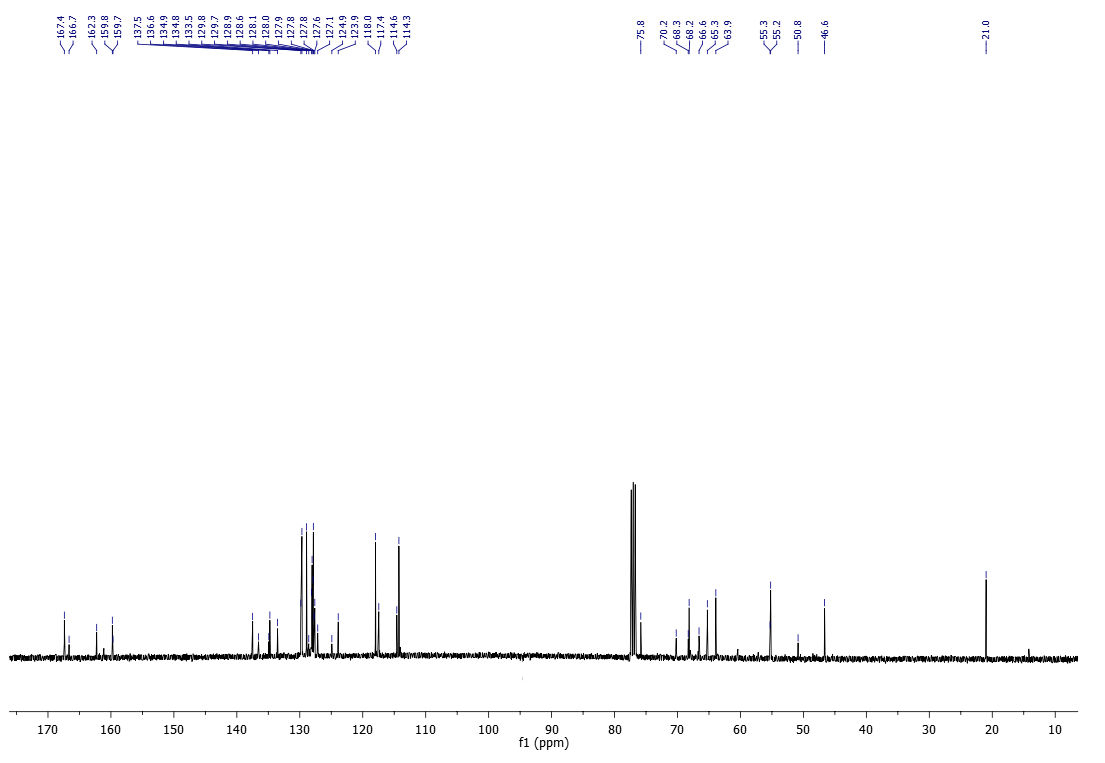


Figure S6. ^13^C NMR spectrum of compound **36** (100 MHz, CDCl_3_).


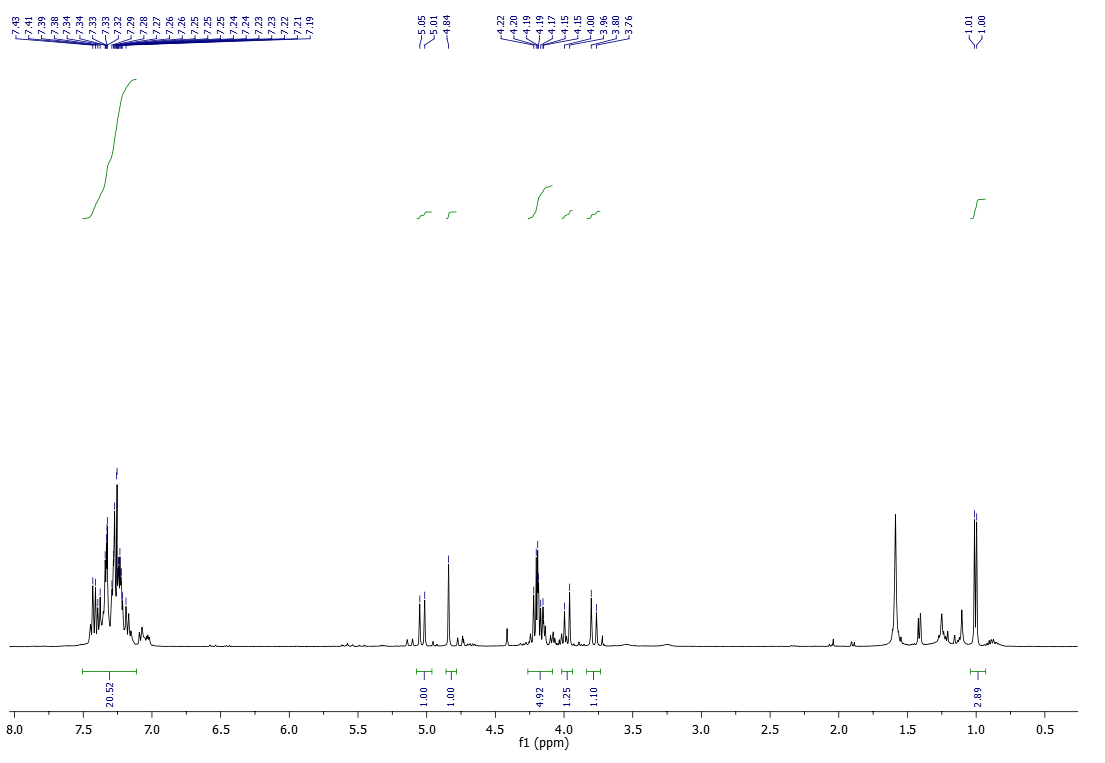


Figure S7. ^1^H NMR spectrum of compound **37** (400 MHz, CDCl_3_).


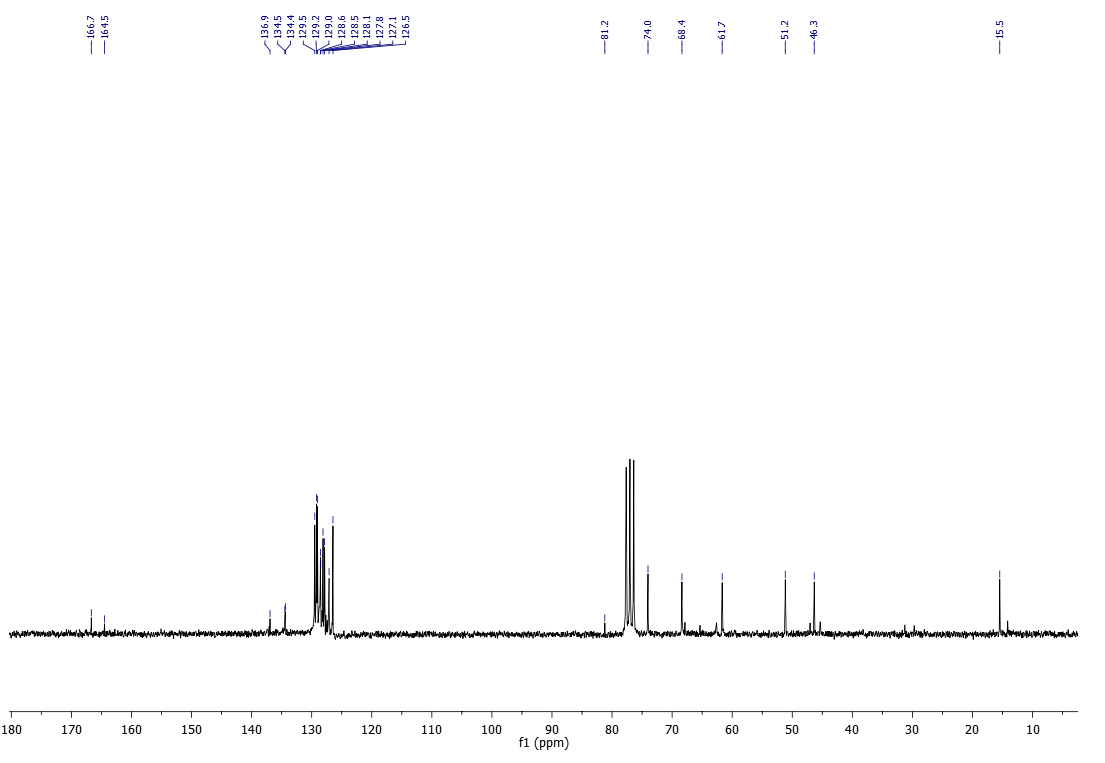
Figure S8. ^13^C NMR spectrum of compound **37** (50 MHz, CDCl_3_).


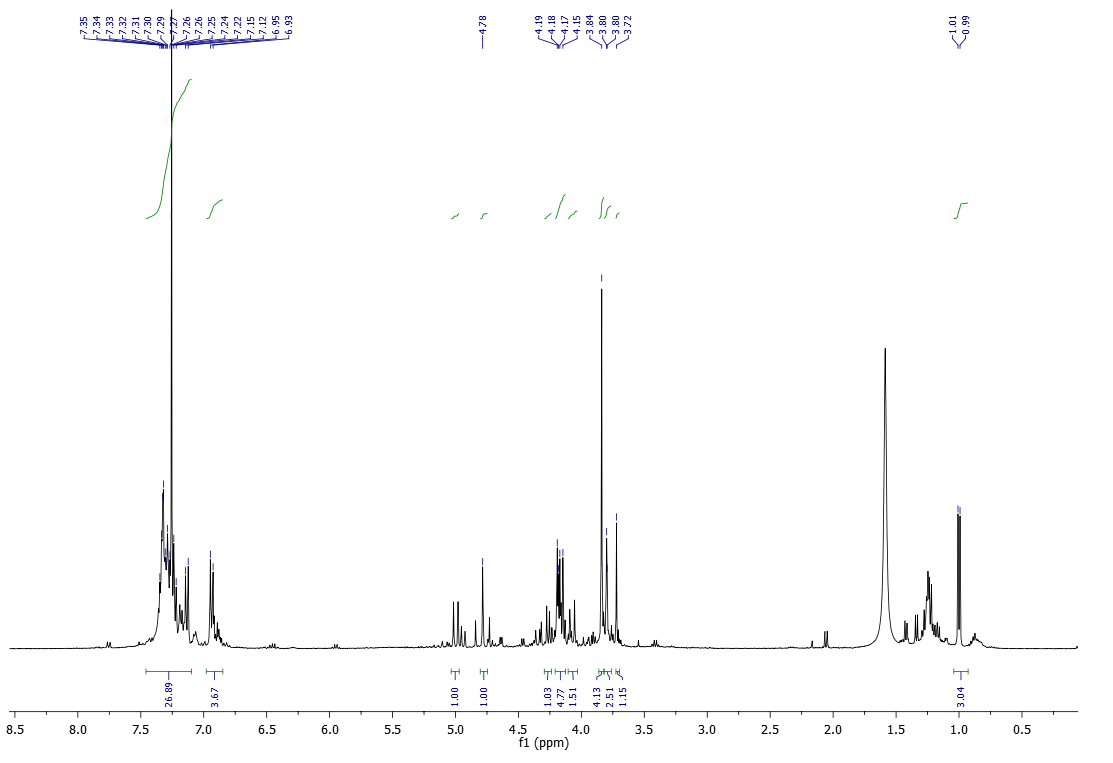
Figure S9. ^1^H NMR spectrum of compound **38** (400 MHz, CDCl_3_).


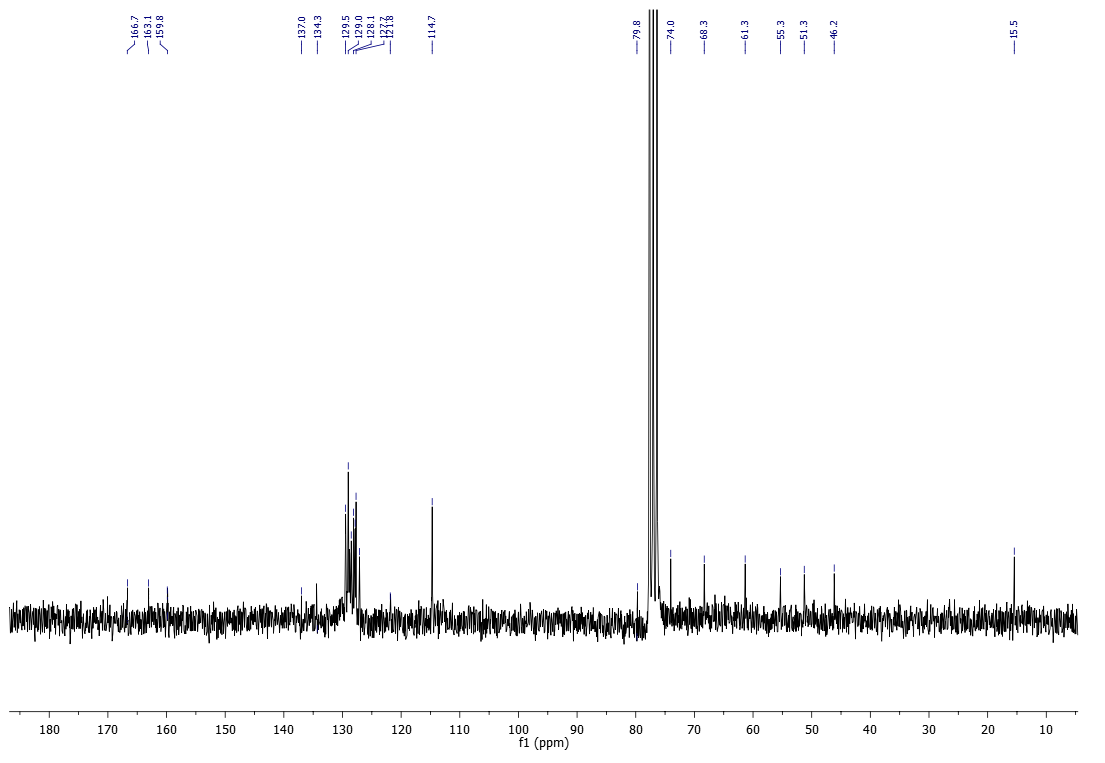
Figure S10. ^13^C NMR spectrum of compound **38** (50 MHz, CDCl_3_).


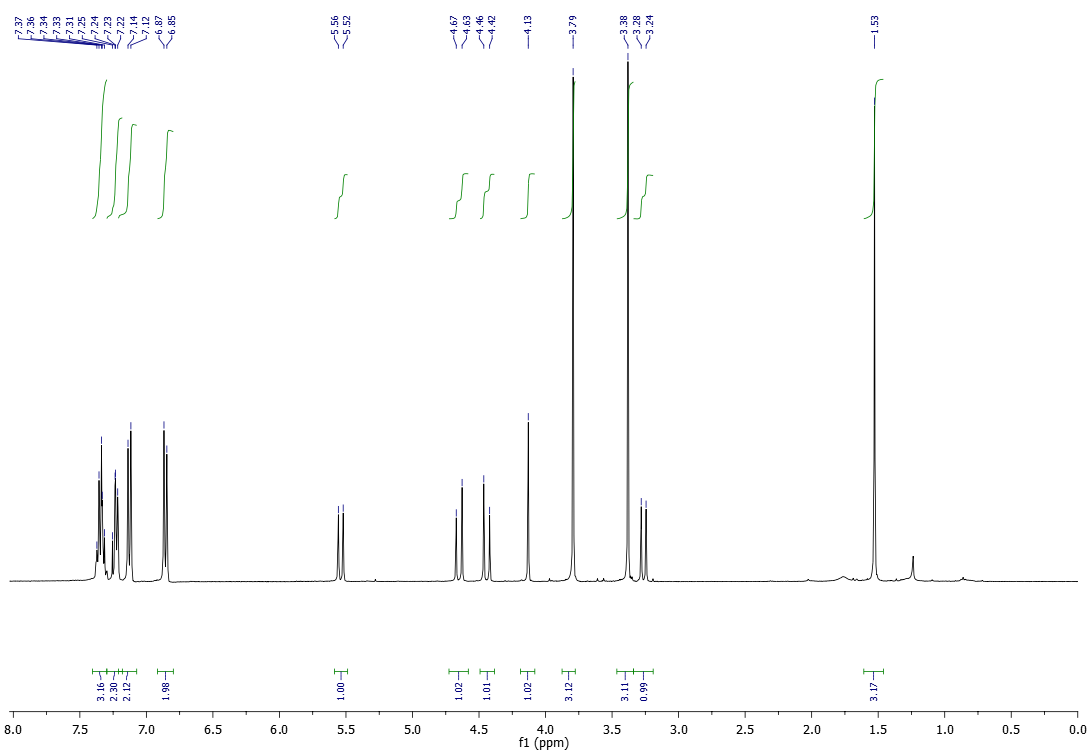


Figure S11. ^1^H NMR spectrum of compound **42** (400 MHz, CDCl_3_).

Figure S12. ^13^C NMR spectrum of compound **42** (100 MHz, CDCl_3_).


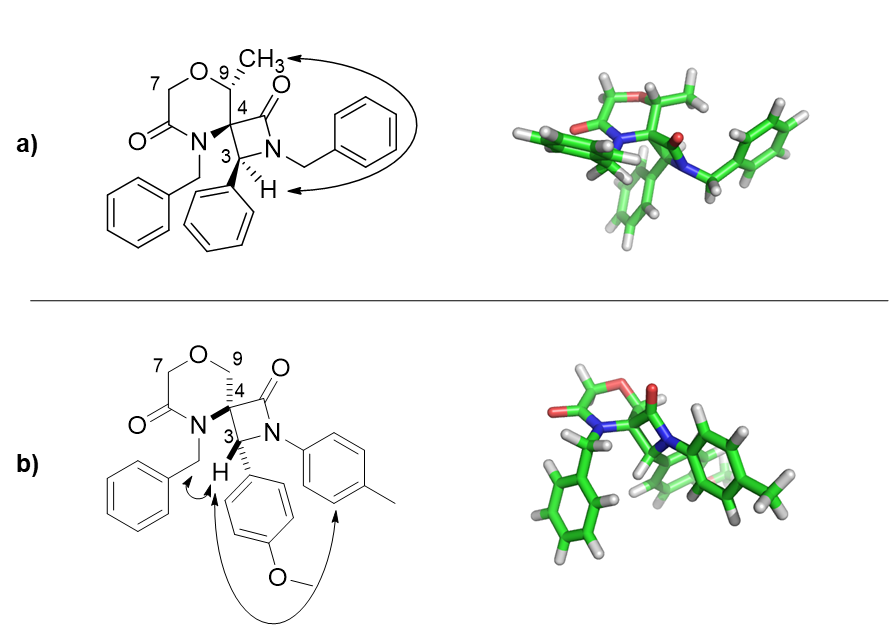


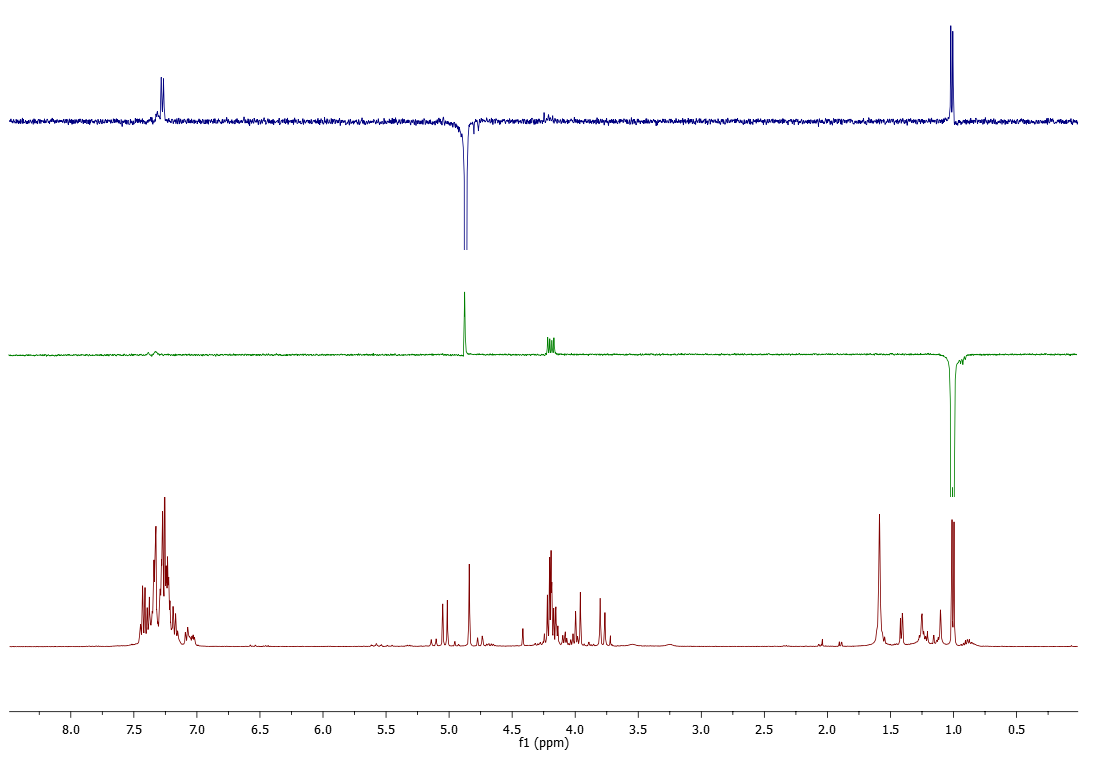


**CH_3_**

**H_3_**

Figure S13. 1D NOESY experiments of compound **37** (400 MHz, CDCl_3_).


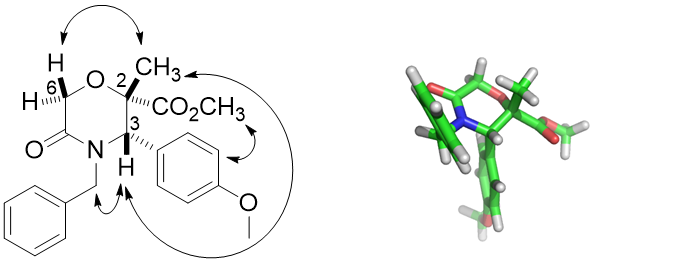


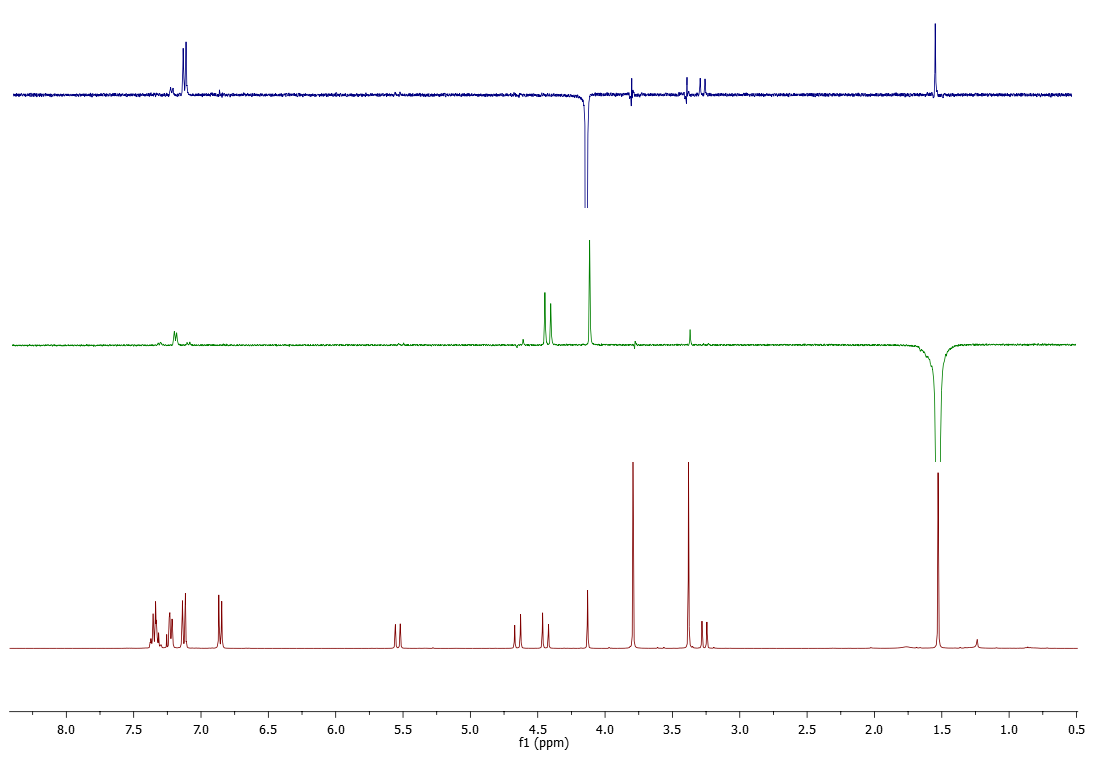


**CH_3_**

**H_6b_**

**H_3_**

Figure S14. 1D NOESY experiments of compound **42** (400 MHz, CDCl_3_).

Table S1. PCA results table for the first three dimensions of morpholine library compounds

| **MOL ID** | **PC1** | **PC2** | **PC3** | **MOL ID** | **PC1** | **PC2** | **PC3** |
| --- | --- | --- | --- | --- | --- | --- | --- |
| **1** | -1,541148 | 0,703663 | -0,086007 | **46** | -2,374855 | -1,158703 | -1,38737 |
| **2** | -1,673943 | 0,857898 | -0,081706 | **47** | -1,295638 | -1,903838 | -1,271803 |
| **3** | -1,326882 | 0,564861 | 0,126071 | **48** | -2,332816 | -1,309781 | -1,423388 |
| **4** | -1,748761 | 0,833539 | -0,405759 | **49** | -0,802913 | 0,212342 | -0,650012 |
| **5** | -1,457219 | 0,603488 | -0,491373 | **50** | -1,209120 | 0,371396 | -0,719512 |
| **6** | -3,154545 | -0,883323 | -1,362029 | **51** | 0,169068 | 1,678186 | 0,254832 |
| **7** | -1,899064 | 0,874189 | -0,538544 | **52** | -1,552757 | 0,601323 | -0,60749 |
| **8** | -2,765563 | -1,084712 | -0,908441 | **53** | -2,572603 | -1,353736 | -0,880827 |
| **9** | -3,121891 | -0,772765 | -1,123044 | **54** | 0,677108 | -2,334505 | 1,827699 |
| **10** | -3,303290 | -0,694739 | -1,294183 | **55** | 2,169964 | -0,987372 | 2,56001 |
| **11** | 0,924511 | 1,713175 | 1,081888 | **56** | -2,943493 | -0,841522 | -1,234982 |
| **12** | -2,323816 | 1,220862 | -0,98588 | **57** | -1,408018 | 0,646606 | -0,385304 |
| **13** | -1,440920 | 0,908810 | -0,719746 | **58** | -0,195732 | 2,126650 | 0,32033 |
| **14** | -2,021046 | 1,059258 | -0,192982 | **59** | -2,803049 | -1,052102 | -1,233467 |
| **15** | 1,264809 | 1,468511 | 1,211567 | **60** | -1,837833 | 2,400944 | 1,34062 |
| **16** | -2,273179 | 1,100533 | -0,430059 | **61** | -1,425658 | 2,202230 | 1,35683 |
| **17** | -2,132978 | 1,082211 | -0,433316 | **62** | -2,906202 | 0,930619 | 0,010582 |
| **18** | 1,295545 | 1,430172 | 1,248242 | **63** | -1,658685 | 2,294502 | 1,507356 |
| **19** | 1,085089 | 1,553441 | 1,045155 | **64** | -0,011746 | -0,231415 | 1,975313 |
| **20** | -3,373783 | -0,752670 | -1,628265 | **65** | 0,352996 | 1,471038 | 2,697839 |
| **21** | -2,765563 | -1,084712 | -0,908441 | **66** | -0,011746 | -0,231415 | 1,975313 |
| **22** | -3,121891 | -0,772765 | -1,123044 | **67** | -1,175564 | 0,227275 | 0,218453 |
| **23** | -3,303290 | -0,694739 | -1,294183 | **68** | -1,175564 | 0,227275 | 0,218453 |
| **24** | -2,980291 | -0,984645 | -1 | **69** | -2 | 0,754003 | 0,407609 |
| **25** | -1,629750 | 0,716595 | -0,726448 | **70** | 0,754635 | 1,454661 | -0,292545 |
| **26** | -3,154545 | -0,883323 | -1,362029 | **71** | -2,075691 | -1,795427 | -2,52569 |
| **27** | 0,303186 | -2,129134 | 1,723852 | **72** | -2,075691 | -1,795427 | -2,52569 |
| **28** | -3,982886 | -0,906091 | -1,648385 | **73** | 1,371461 | 1,030234 | -0,600329 |
| **29** | -1,738391 | 0,773283 | -0,229001 | **74** | 1,371461 | 1,030234 | -0,600329 |
| **30** | 1,280398 | 1,657812 | 1,118252 | **75** | 1,127785 | 1,311264 | -0,241421 |
| **31** | -2,101513 | 0,954635 | -0,511396 | **76** | 0,529496 | 1,618797 | 0,113817 |
| **32** | -1,627498 | 0,572040 | 0,221623 | **77** | -0,413946 | -0,057156 | -1,270597 |
| **33** | -1,430255 | 0,587557 | -0,028643 | **78** | -0,287804 | 0,065299 | -1,325948 |
| **34** | -0,836996 | 0,204097 | -0,604393 | **79** | 0,529496 | 1,618797 | 0,113817 |
| **35** | -0,974778 | 0,384463 | -0,670643 | **80** | -0,064027 | 1,930090 | 0,457406 |
| **36** | 0,131412 | 1,648945 | -0,025491 | **81** | -3,750377 | -1,287016 | -1,208018 |
| **37** | -0,856358 | 0,199218 | -0,33756 | **82** | -3,750377 | -1,287016 | -1,208018 |
| **38** | -0,874496 | 0,231036 | -0,650953 | **83** | 0,026717 | 1,851641 | 1,113443 |
| **39** | -2,996516 | -1,110731 | -1,420084 | **84** | 0,026717 | 1,851641 | 1,113443 |
| **40** | -2,396483 | -1,393159 | -0,718584 | **85** | 0,000943 | 1,916311 | 0,54782 |
| **41** | -2,396483 | -1,393159 | -0,718584 | **86** | 0,000943 | 1,916311 | 0,54782 |
| **42** | -2,782279 | -1,201664 | -1,164716 | **87** | -0,410413 | 2,087265 | 0,530338 |
| **43** | -2,179744 | -1,488253 | -1,5159 | **88** | -0,378954 | 2,042821 | 1,030643 |
| **44** | -2,297971 | -1,363165 | -1,379629 | **89** | -0,543452 | 2,227892 | 0,679915 |
| **45** | -2,100961 | -1,570792 | -1,119375 | **90** | -0,558560 | 2,135844 | 0,863627 |
| **MOL ID** | **PC1** | **PC2** | **PC3** | **MOL ID** | **PC1** | **PC2** | **PC3** |
| **91** | -0,369325 | 2,108996 | 0,91532 | **138** | -0,455297 | 0,010440 | -0,21298 |
| **92** | -0,582456 | 2,177927 | 0,298028 | **139** | -0,455297 | 0,010440 | -0,21298 |
| **93** | -3,313834 | -1,483947 | -1,800951 | **140** | -0,238871 | -1,948030 | 0,376387 |
| **94** | -2,265485 | 0,697538 | -0,192886 | **141** | 0,808030 | -0,269056 | 0,903508 |
| **95** | -1,513659 | 0,487401 | 0,108589 | **142** | -0,238871 | -1,948030 | 0,376387 |
| **96** | -1,903117 | 0,756591 | -0,085481 | **143** | 0,80803 | -0,269056 | 0,903508 |
| **97** | -0,388999 | 0,270821 | 0,62538 | **144** | -1,344446 | -1,744622 | -0,381875 |
| **98** | -0,782259 | 0,425310 | 0,056891 | **145** | -0,381033 | 0,321677 | 0,155799 |
| **99** | -0,782259 | 0,425310 | 0,056891 | **146** | -1,011561 | -1,627569 | 0,108149 |
| **100** | -1,262152 | 0,470356 | -0,489816 | **147** | -1,011561 | -1,627569 | 0,108149 |
| **101** | -1,004004 | 0,581114 | -0,729906 | **148** | -0,258091 | -2,054034 | -0,331763 |
| **102** | -0,434141 | 0,266388 | 0,421007 | **149** | -0,381033 | 0,321677 | 0,155799 |
| **103** | -0,434141 | 0,266388 | 0,421007 | **150** | -1,044101 | -1,588803 | 0,047684 |
| **104** | 6,254258 | -0,096509 | 1,622724 | **151** | -1,044101 | -1,588803 | 0,047684 |
| **105** | 0,510325 | -2,207885 | -0,748006 | **152** | -1,044112 | -1,591268 | 0,03649 |
| **106** | 2,598080 | -2,641528 | -0,62069 | **153** | -1,044112 | -1,591268 | 0,03649 |
| **107** | 5,138126 | -3,131111 | 0,084342 | **154** | -1,249281 | -1,480034 | -0,233797 |
| **108** | 2,018787 | 2,852832 | 1,717214 | **155** | -1,067063 | -1,574469 | -0,527156 |
| **109** | 2,018787 | 2,852832 | 1,717214 | **156** | -1,067063 | -1,574469 | -0,527156 |
| **110** | 0,950453 | 3,232186 | 1,520721 | **157** | -1,067063 | -1,574469 | -0,527156 |
| **111** | 0,950453 | 3,232186 | 1,520721 | **158** | -0,174142 | 1,683570 | 0,419277 |
| **112** | 0,923085 | 3,290001 | 1,565615 | **159** | -1,249281 | -1,480034 | -0,233797 |
| **113** | 0,923085 | 3,290001 | 1,565615 | **160** | -0,174142 | 0,168357 | 0,419277 |
| **114** | 1,152677 | 3,210109 | 1,38018 | **161** | -1,250215 | 0,532335 | 0,1438 |
| **115** | 1,152677 | 3,210109 | 1,38018 | **162** | -1,344446 | -1,744622 | -0,381875 |
| **116** | 1,548761 | 3,299216 | 1,020903 | **163** | -0,455297 | 0,010440 | -0,21298 |
| **117** | 1,548761 | 3,299216 | 1,020903 | **164** | -0,763072 | 0,373413 | -1,0441 |
| **118** | 1,732055 | 3,211549 | 1,20781 | **165** | -1,889387 | 0,771838 | 0,024009 |
| **119** | 1,732055 | 3,211549 | 1,20781 | **166** | 0,022086 | 1,967984 | 0,831041 |
| **120** | 1,336367 | 3,119610 | 1,567822 | **167** | -2,129748 | 0,958687 | -0,049214 |
| **121** | 1,336367 | 3,119610 | 1,567822 | **168** | -0,541880 | 2,142237 | 1,128283 |
| **122** | 1,535093 | 3,306388 | 1,657321 | **169** | 1,318122 | 1,501511 | 1,506345 |
| **123** | 1,535093 | 3,306388 | 1,657321 | **170** | 1,318122 | 1,501511 | 1,506345 |
| **124** | 0,342290 | 1,696030 | 1,325281 | **171** | -0,541880 | 2,142237 | 1,128283 |
| **125** | 0,144714 | 1,933901 | 0,165979 | **172** | -1,328651 | 0,654633 | 0,073511 |
| **126** | -0,029222 | 2,018058 | 0,457172 | **173** | 0,263151 | 1,818488 | 1,256332 |
| **127** | 1,123306 | 1,222406 | 0,96056 | **174** | -0,268250 | 0,299988 | 0,4057 |
| **128** | 0,310238 | -0,324459 | 0,597176 | **175** | 0,263151 | 1,818488 | 1,256332 |
| **129** | 0,749764 | 1,502643 | 0,750277 | **176** | -2,251386 | 0,782214 | -0,141662 |
| **130** | 0,533689 | 1,527139 | 0,31202 | **177** | -2,420987 | 0,869918 | -0,370114 |
| **131** | 1,162224 | 1,263843 | 0,848412 | **178** | -0,640925 | 2,025866 | 0,674857 |
| **132** | 1,123306 | 1,222406 | 0,96056 | **179** | 0,786008 | 3,231783 | 1,584825 |
| **133** | -0,411259 | 0,197816 | -0,570115 | **180** | 1,193538 | 3,032230 | 1,656075 |
| **134** | -0,411259 | 0,197816 | -0,570115 | **181** | -0,462198 | 1,925783 | 0,812086 |
| **135** | -1,250215 | 0,532335 | 0,1438 | **182** | 0,986161 | 3,182679 | 1,533474 |
| **136** | -0,455297 | 0,010440 | -0,21298 | **183** | 0,606074 | 3,327628 | 1,416935 |
| **137** | -0,455297 | 0,010440 | -0,21298 |  |  |  |  |

Table S2: PMI results table for morpholine library compounds

| **MOLID** | **I1** | **I2** | **I3** | **I2/I3** | **I1/I3** |
| --- | --- | --- | --- | --- | --- |
| **1** | 3687,9539 | 3218,9777 | 840,0751 | 0,8728 | 0,2278 |
| **2** | 3768,9034 | 3137,2321 | 759,5119 | 0,8324 | 0,2015 |
| **3** | 4332,0202 | 3754,7247 | 975,3587 | 0,8667 | 0,2252 |
| **4** | 3037,1785 | 2646,7818 | 771,1435 | 0,8715 | 0,2539 |
| **5** | 4785,2122 | 4399,8300 | 692,7546 | 0,9195 | 0,1448 |
| **6** | 1351,4185 | 1049,0133 | 489,6340 | 0,7762 | 0,3623 |
| **7** | 2912,2702 | 2642,0997 | 624,9829 | 0,9072 | 0,2146 |
| **8** | 2078,5661 | 1839,6145 | 519,2793 | 0,8850 | 0,2498 |
| **9** | 1568,1641 | 1114,5588 | 529,4317 | 0,7107 | 0,3376 |
| **10** | 1399,7814 | 990,5079 | 446,0086 | 0,7076 | 0,3186 |
| **11** | 4930,5006 | 4447,7576 | 3013,7260 | 0,9021 | 0,6112 |
| **12** | 1792,7004 | 1478,2408 | 572,6464 | 0,8246 | 0,3194 |
| **13** | 1928,9406 | 1870,6355 | 1106,3415 | 0,9698 | 0,5735 |
| **14** | 2563,0310 | 2007,0581 | 1535,8460 | 0,7831 | 0,5992 |
| **15** | 11387,0352 | 10357,4469 | 2241,9841 | 0,9096 | 0,1969 |
| **16** | 1945,6743 | 1652,8016 | 682,5330 | 0,8495 | 0,3508 |
| **17** | 2971,0160 | 2801,3274 | 639,4227 | 0,9429 | 0,2152 |
| **18** | 12617,9096 | 11337,7914 | 2313,6352 | 0,8985 | 0,1834 |
| **19** | 9814,4499 | 8717,1648 | 2292,2736 | 0,8882 | 0,2336 |
| **20** | 1108,9919 | 793,0910 | 450,8365 | 0,7151 | 0,4065 |
| **21** | 1857,1868 | 1659,6375 | 609,4986 | 0,8936 | 0,3282 |
| **22** | 1566,4503 | 1084,0932 | 548,0793 | 0,6921 | 0,3499 |
| **23** | 1382,4894 | 989,1165 | 437,8535 | 0,7155 | 0,3167 |
| **24** | 1439,5274 | 1223,6698 | 577,4025 | 0,8500 | 0,4011 |
| **25** | 4415,3218 | 3990,5536 | 619,8411 | 0,9038 | 0,1404 |
| **26** | 1337,7401 | 1013,7253 | 532,4362 | 0,7578 | 0,3980 |
| **27** | 7185,6404 | 4232,7957 | 3946,7794 | 0,5891 | 0,5493 |
| **28** | 970,3609 | 834,5247 | 220,8237 | 0,8600 | 0,2276 |
| **29** | 3593,0062 | 2923,6212 | 1212,5852 | 0,8137 | 0,3375 |
| **30** | 10308,3892 | 9619,6573 | 2449,0946 | 0,9332 | 0,2376 |
| **31** | 2477,9849 | 1709,7083 | 1106,9982 | 0,6900 | 0,4467 |
| **32** | 5046,4814 | 4483,9397 | 1006,4940 | 0,8885 | 0,1994 |
| **33** | 3609,9028 | 2961,2303 | 1029,4193 | 0,8203 | 0,2852 |
| **34** | 3420,5445 | 2438,6616 | 1619,4069 | 0,7129 | 0,4734 |
| **35** | 3206,1511 | 2450,7139 | 1428,3789 | 0,7644 | 0,4455 |
| **36** | 4509,8483 | 3625,2336 | 2434,6164 | 0,8038 | 0,5398 |
| **37** | 3203,8903 | 2777,1507 | 1400,1418 | 0,8668 | 0,4370 |
| **38** | 2924,7317 | 2634,5005 | 1282,6898 | 0,9008 | 0,4386 |
| **39** | 1789,6739 | 1434,9019 | 510,5240 | 0,8018 | 0,2853 |
| **40** | 2718,1474 | 2404,7854 | 676,2068 | 0,8847 | 0,2488 |
| **41** | 2817,2021 | 2366,8868 | 789,2632 | 0,8402 | 0,2802 |
| **42** | 1848,5327 | 1585,6237 | 668,2134 | 0,8578 | 0,3615 |
| **43** | 2293,1786 | 1942,9398 | 800,2006 | 0,8473 | 0,3489 |
| **44** | 2242,7709 | 2010,6636 | 684,3002 | 0,8965 | 0,3051 |
| **45** | 2791,5471 | 2546,7618 | 716,2016 | 0,9123 | 0,2566 |
| **46** | 2212,2783 | 1968,3745 | 674,3745 | 0,8897 | 0,3048 |
| **47** | 3972,2083 | 3589,5218 | 970,4625 | 0,9037 | 0,2443 |
| **48** | 2181,9725 | 1850,7071 | 686,0280 | 0,8482 | 0,3144 |
| **49** | 5091,5742 | 4714,4974 | 1110,0860 | 0,9259 | 0,2180 |
| **50** | 3765,4731 | 3551,4898 | 885,8059 | 0,9432 | 0,2352 |
| **51** | 8000,4924 | 7590,2075 | 1325,7714 | 0,9487 | 0,1657 |
| **52** | 2960,4957 | 2665,5457 | 934,7268 | 0,9004 | 0,3157 |
| **53** | 2174,6743 | 1854,4260 | 872,0669 | 0,8527 | 0,4010 |
| **54** | 6507,7621 | 5146,9969 | 3963,5053 | 0,7909 | 0,6090 |
| **55** | 10993,8826 | 8348,8741 | 4886,7377 | 0,7594 | 0,4445 |
| **56** | 1478,2433 | 1307,2820 | 479,2031 | 0,8843 | 0,3242 |
| **57** | 4265,0349 | 3836,3821 | 929,6140 | 0,8995 | 0,2180 |
| **58** | 5495,0271 | 4629,2342 | 1539,1636 | 0,8424 | 0,2801 |
| **59** | 1683,4949 | 1364,9529 | 570,7771 | 0,8108 | 0,3390 |
| **60** | 2690,4892 | 2329,8784 | 815,4313 | 0,8660 | 0,3031 |
| **61** | 4601,7059 | 3400,4579 | 1522,1197 | 0,7390 | 0,3308 |
| **62** | 3072,8346 | 3063,1321 | 218,3817 | 0,9968 | 0,0711 |
| **63** | 2924,4376 | 2360,9128 | 963,9547 | 0,8073 | 0,3296 |
| **64** | 6162,1351 | 5185,0159 | 2664,6254 | 0,8414 | 0,4324 |
| **65** | 7928,2980 | 7610,3305 | 1636,7961 | 0,9599 | 0,2064 |
| **66** | 6188,4322 | 4258,6989 | 3257,0359 | 0,6882 | 0,5263 |
| **67** | 3867,1280 | 3023,7546 | 1667,2125 | 0,7819 | 0,4311 |
| **68** | 4036,7206 | 3416,7329 | 1823,6275 | 0,8464 | 0,4518 |
| **69** | 3687,1667 | 2932,5217 | 1286,7346 | 0,7953 | 0,3490 |
| **70** | 4137,7904 | 3515,0956 | 2259,5857 | 0,8495 | 0,5461 |
| **71** | 1671,3558 | 1485,4349 | 1017,5281 | 0,8888 | 0,6088 |
| **72** | 2422,0072 | 1866,9608 | 794,2956 | 0,7708 | 0,3279 |
| **73** | 4502,5252 | 4005,8908 | 2662,0039 | 0,8897 | 0,5912 |
| **74** | 5197,2627 | 4631,5704 | 2598,8087 | 0,8912 | 0,5000 |
| **75** | 6991,0727 | 5532,5168 | 2532,6802 | 0,7914 | 0,3623 |
| **76** | 5091,1025 | 4555,6336 | 2143,6168 | 0,8948 | 0,4211 |
| **77** | 5285,4117 | 3810,7007 | 1757,1931 | 0,7210 | 0,3325 |
| **78** | 3925,6514 | 3065,0933 | 1635,1018 | 0,7808 | 0,4165 |
| **79** | 6264,9089 | 5126,6102 | 1961,8739 | 0,8183 | 0,3132 |
| **80** | 6460,0803 | 5455,9793 | 1675,7432 | 0,8446 | 0,2594 |
| **81** | 1067,5211 | 1012,9499 | 351,4320 | 0,9489 | 0,3292 |
| **82** | 1028,9004 | 1012,6541 | 313,1851 | 0,9842 | 0,3044 |
| **83** | 7222,7188 | 6068,6339 | 1697,1183 | 0,8402 | 0,2350 |
| **84** | 4745,6724 | 4471,8125 | 1807,5103 | 0,9423 | 0,3809 |
| **85** | 6758,5333 | 5863,5692 | 1408,9803 | 0,8676 | 0,2085 |
| **86** | 4578,5605 | 4449,1970 | 1683,2001 | 0,9717 | 0,3676 |
| **87** | 4972,0421 | 4365,0369 | 1279,4115 | 0,8779 | 0,2573 |
| **88** | 5442,5604 | 4657,3352 | 1447,9879 | 0,8557 | 0,2660 |
| **89** | 5059,2744 | 4537,3960 | 1429,6759 | 0,8968 | 0,2826 |
| **90** | 4190,0523 | 3708,3133 | 1611,2092 | 0,8850 | 0,3845 |
| **91** | 5409,2155 | 4824,7041 | 1598,6966 | 0,8919 | 0,2956 |
| **92** | 4751,2609 | 4074,4073 | 1480,9383 | 0,8575 | 0,3117 |
| **93** | 1440,2412 | 1112,6892 | 502,8275 | 0,7726 | 0,3491 |
| **94** | 2135,6068 | 1877,1021 | 716,2570 | 0,8790 | 0,3354 |
| **95** | 4393,6496 | 3706,4406 | 1291,7096 | 0,8436 | 0,2940 |
| **96** | 3094,4388 | 2737,3291 | 850,4263 | 0,8846 | 0,2748 |
| **97** | 5515,5785 | 3833,6505 | 2724,8266 | 0,6951 | 0,4940 |
| **98** | 4941,6168 | 3508,3597 | 2077,7478 | 0,7100 | 0,4205 |
| **99** | 4938,6338 | 4261,2644 | 1529,3661 | 0,8628 | 0,3097 |
| **100** | 4195,2739 | 3547,9174 | 1052,2037 | 0,8457 | 0,2508 |
| **101** | 4461,2442 | 3687,8662 | 1212,0217 | 0,8266 | 0,2717 |
| **102** | 5646,7350 | 3593,8222 | 2785,0444 | 0,6364 | 0,4932 |
| **103** | 5742,5056 | 5013,8752 | 1794,8574 | 0,8731 | 0,3126 |
| **104** | 14522,7211 | 12360,2188 | 2162,5023 | 0,8511 | 0,1489 |
| **105** | 5299,7049 | 3779,1283 | 2723,1479 | 0,7131 | 0,5138 |
| **106** | 9041,4106 | 8075,3650 | 3472,6839 | 0,8932 | 0,3841 |
| **107** | 11498,8993 | 10514,5351 | 984,3641 | 0,9144 | 0,0856 |
| **108** | 9482,3007 | 8722,8828 | 3122,1947 | 0,9199 | 0,3293 |
| **109** | 8151,1141 | 6855,8264 | 3017,8240 | 0,8411 | 0,3702 |
| **110** | 6288,5409 | 5759,6948 | 2104,5759 | 0,9159 | 0,3347 |
| **111** | 6381,3838 | 5175,6150 | 2040,3076 | 0,8110 | 0,3197 |
| **112** | 7116,1894 | 6865,5079 | 1968,4787 | 0,9648 | 0,2766 |
| **113** | 7398,3392 | 6393,9047 | 1642,0370 | 0,8642 | 0,2219 |
| **114** | 7987,5348 | 7861,3103 | 2093,6151 | 0,9842 | 0,2621 |
| **115** | 8432,5209 | 7376,0477 | 1710,5392 | 0,8747 | 0,2029 |
| **116** | 9014,2528 | 8748,4063 | 1970,2298 | 0,9705 | 0,2186 |
| **117** | 9324,4084 | 8331,6857 | 1700,1070 | 0,8935 | 0,1823 |
| **118** | 8530,6975 | 6882,6521 | 3283,3934 | 0,8068 | 0,3849 |
| **119** | 8028,3494 | 6287,5794 | 2763,9835 | 0,7832 | 0,3443 |
| **120** | 8241,1956 | 7022,2993 | 2969,7083 | 0,8521 | 0,3603 |
| **121** | 8070,2858 | 6339,0600 | 2384,8154 | 0,7855 | 0,2955 |
| **122** | 9738,6786 | 7188,1294 | 4272,8461 | 0,7381 | 0,4388 |
| **123** | 9200,3617 | 6388,6241 | 3485,2776 | 0,6944 | 0,3788 |
| **124** | 7327,7926 | 6709,5081 | 1368,5268 | 0,9156 | 0,1868 |
| **125** | 8873,9049 | 8575,0266 | 1162,2635 | 0,9663 | 0,1310 |
| **126** | 8929,5167 | 8580,5231 | 1147,2683 | 0,9609 | 0,1285 |
| **127** | 7254,3983 | 6900,7839 | 2274,5040 | 0,9513 | 0,3135 |
| **128** | 6199,5131 | 5473,6320 | 2360,2734 | 0,8829 | 0,3807 |
| **129** | 7652,9356 | 7197,0299 | 1633,2706 | 0,9404 | 0,2134 |
| **130** | 5714,7102 | 4810,8995 | 1739,5038 | 0,8418 | 0,3044 |
| **131** | 10269,5866 | 9412,7450 | 1798,0921 | 0,9166 | 0,1751 |
| **132** | 7831,7072 | 7050,3852 | 2006,4317 | 0,9002 | 0,2562 |
| **133** | 4565,0698 | 4075,4215 | 1410,2967 | 0,8927 | 0,3089 |
| **134** | 4975,6295 | 4191,0785 | 1508,7389 | 0,8423 | 0,3032 |
| **135** | 4483,3003 | 4362,5168 | 889,2299 | 0,9731 | 0,1983 |
| **136** | 5267,3094 | 4778,1296 | 1416,2668 | 0,9071 | 0,2689 |
| **137** | 5336,2426 | 4776,4364 | 1358,2268 | 0,8951 | 0,2545 |
| **138** | 5545,6957 | 5023,3447 | 1310,9756 | 0,9058 | 0,2364 |
| **139** | 8519,8409 | 7704,7309 | 1801,5949 | 0,9043 | 0,2115 |
| **140** | 6423,1582 | 5714,5392 | 1827,9495 | 0,8897 | 0,2846 |
| **141** | 10099,2427 | 8209,6450 | 2923,8759 | 0,8129 | 0,2895 |
| **142** | 5577,9001 | 4915,8876 | 1818,9732 | 0,8813 | 0,3261 |
| **143** | 8547,8077 | 5258,6226 | 4146,4515 | 0,6152 | 0,4851 |
| **144** | 4561,4955 | 4364,8489 | 954,9873 | 0,9569 | 0,2094 |
| **145** | 5572,7402 | 4932,4221 | 1434,5556 | 0,8851 | 0,2574 |
| **146** | 5367,1957 | 4973,8475 | 1072,5315 | 0,9267 | 0,1998 |
| **147** | 5391,2564 | 5104,3134 | 1025,7867 | 0,9468 | 0,1903 |
| **148** | 6297,1797 | 5863,4764 | 1525,9177 | 0,9311 | 0,2423 |
| **149** | 5769,0064 | 5216,1607 | 1319,9981 | 0,9042 | 0,2288 |
| **150** | 4780,4756 | 4370,9121 | 1328,7988 | 0,9143 | 0,2780 |
| **151** | 5047,3456 | 4714,1446 | 1149,4093 | 0,9340 | 0,2277 |
| **152** | 5317,6086 | 4856,5360 | 1189,3844 | 0,9133 | 0,2237 |
| **153** | 5162,4546 | 4806,1538 | 1082,7912 | 0,9310 | 0,2097 |
| **154** | 4359,1360 | 3939,0743 | 1168,1797 | 0,9036 | 0,2680 |
| **155** | 5075,3739 | 4902,9792 | 991,7015 | 0,9660 | 0,1954 |
| **156** | 5258,5522 | 5116,8611 | 866,4469 | 0,9731 | 0,1648 |
| **157** | 5064,7003 | 4805,4970 | 982,3071 | 0,9488 | 0,1940 |
| **158** | 7149,2798 | 6746,5042 | 1157,9004 | 0,9437 | 0,1620 |
| **159** | 4649,4136 | 4360,3355 | 1098,0235 | 0,9378 | 0,2362 |
| **160** | 7285,3471 | 7036,5128 | 1046,2068 | 0,9658 | 0,1436 |
| **161** | 4986,9630 | 4780,6636 | 670,4954 | 0,9586 | 0,1344 |
| **162** | 4200,0938 | 3909,1568 | 1025,6207 | 0,9307 | 0,2442 |
| **163** | 5631,0578 | 5157,9538 | 1184,4235 | 0,9160 | 0,2103 |
| **164** | 5365,4990 | 5196,9444 | 848,3370 | 0,9686 | 0,1581 |
| **165** | 3275,6064 | 3034,7913 | 786,5054 | 0,9265 | 0,2401 |
| **166** | 5648,2013 | 4916,4861 | 1405,1008 | 0,8705 | 0,2488 |
| **167** | 3097,4056 | 2777,2800 | 673,7952 | 0,8966 | 0,2175 |
| **168** | 5001,4262 | 4327,4397 | 1350,5616 | 0,8652 | 0,2700 |
| **169** | 12691,7876 | 11715,4103 | 1997,0804 | 0,9231 | 0,1574 |
| **170** | 12020,1199 | 10807,3728 | 2126,0653 | 0,8991 | 0,1769 |
| **171** | 5001,9475 | 4325,7798 | 1352,7833 | 0,8648 | 0,2705 |
| **172** | 5031,6650 | 4915,3449 | 821,6668 | 0,9769 | 0,1633 |
| **173** | 7614,5944 | 6965,1785 | 1529,8801 | 0,9147 | 0,2009 |
| **174** | 9563,7693 | 9391,7908 | 914,9864 | 0,9820 | 0,0957 |
| **175** | 7369,3955 | 6792,9723 | 1550,2257 | 0,9218 | 0,2104 |
| **176** | 1985,0424 | 1487,9800 | 875,2570 | 0,7496 | 0,4409 |
| **177** | 1776,9980 | 1316,5519 | 807,0495 | 0,7409 | 0,4542 |
| **178** | 3874,1123 | 3572,6012 | 1833,5168 | 0,9222 | 0,4733 |
| **179** | 4873,1862 | 4556,7979 | 2253,1369 | 0,9351 | 0,4624 |
| **180** | 5789,1978 | 4871,9413 | 3101,7440 | 0,8416 | 0,5358 |
| **181** | 3888,3042 | 3716,1665 | 1835,5895 | 0,9557 | 0,4721 |
| **182** | 6881,4683 | 4535,6947 | 3099,5348 | 0,6591 | 0,4504 |
| **183** | 4846,0621 | 4376,6991 | 2081,2667 | 0,9031 | 0,4295 |

Table S3: Medicinally-relevant molecular properties (MW, ClogP, HBD, HBA, rotatable bonds count, Fsp^3^ and FC*) for morpholine library compounds

| **MOLID** | **MW** | **logP** | **HBD** | **HBA** | **Fsp^3^** | **FC*** | **Rot. Bonds** |
| --- | --- | --- | --- | --- | --- | --- | --- |
| **1** | 300.35 | 2.23 | 1 | 5 | 0,41 | 0,18 | 2 |
| **2** | 298.34 | 1.57 | 0 | 5 | 0,41 | 0,18 | 1 |
| **3** | 314.38 | 2.58 | 1 | 5 | 0,44 | 0,17 | 3 |
| **4** | 286.33 | 1.45 | 1 | 5 | 0,38 | 0,13 | 1 |
| **5** | 302.33 | 0.61 | 1 | 6 | 0,38 | 0,19 | 4 |
| **6** | 210.23 | 0.16 | 1 | 5 | 0,60 | 0,20 | 1 |
| **7** | 272.3 | 1.22 | 1 | 5 | 0,33 | 0,20 | 2 |
| **8** | 238.28 | 0.95 | 1 | 5 | 0,67 | 0,25 | 2 |
| **9** | 222.24 | -0.07 | 0 | 5 | 0,64 | 0,27 | 0 |
| **10** | 208.21 | -0.5 | 0 | 5 | 0,60 | 0,20 | 0 |
| **11** | 462.49 | 3.13 | 0 | 8 | 0,35 | 0,12 | 6 |
| **12** | 244.25 | 0.72 | 1 | 5 | 0,23 | 0,15 | 0 |
| **13** | 306.27 | 1.48 | 0 | 8 | 0,29 | 0,14 | 4 |
| **14** | 387.17 | 2.31 | 0 | 5 | 0,29 | 0,14 | 3 |
| **15** | 478.54 | 4.23 | 1 | 8 | 0,37 | 0,15 | 8 |
| **16** | 259.26 | 1.37 | 0 | 5 | 0,29 | 0,14 | 2 |
| **17** | 275.26 | 1.3 | 0 | 6 | 0,29 | 0,14 | 3 |
| **18** | 478.54 | 4.15 | 1 | 8 | 0,37 | 0,11 | 9 |
| **19** | 464.51 | 3.79 | 1 | 8 | 0,35 | 0,12 | 8 |
| **20** | 196.2 | -0.62 | 1 | 5 | 0,56 | 0,11 | 0 |
| **21** | 238.28 | 0.95 | 1 | 5 | 0,67 | 0,25 | 2 |
| **22** | 222.24 | -0.07 | 0 | 5 | 0,64 | 0,27 | 0 |
| **23** | 208.21 | -0.5 | 0 | 5 | 0,60 | 0,20 | 0 |
| **24** | 224.26 | 0.59 | 1 | 5 | 0,64 | 0,27 | 1 |
| **25** | 288.3 | 0.17 | 1 | 6 | 0,33 | 0,13 | 4 |
| **26** | 210.23 | 0.16 | 1 | 5 | 0,60 | 0,20 | 1 |
| **27** | 448.6 | 5.7 | 0 | 7 | 0,80 | 0,12 | 15 |
| **28** | 173.17 | -0.77 | 1 | 5 | 0,86 | 0,43 | 1 |
| **29** | 307.3 | 1.06 | 0 | 7 | 0,47 | 0,20 | 4 |
| **30** | 492.52 | 2.73 | 0 | 9 | 0,44 | 0,19 | 5 |
| **31** | 277.27 | 0.82 | 0 | 6 | 0,43 | 0,21 | 2 |
| **32** | 319.35 | 1.31 | 0 | 6 | 0,59 | 0,35 | 4 |
| **33** | 316.35 | 0.98 | 0 | 6 | 0,53 | 0,18 | 3 |
| **34** | 334.37 | 0.43 | 2 | 7 | 0,41 | 0,18 | 6 |
| **35** | 332.35 | -0.1 | 2 | 7 | 0,41 | 0,18 | 5 |
| **36** | 384.43 | 1.28 | 2 | 7 | 0,33 | 0,14 | 6 |
| **37** | 348.39 | 0.46 | 1 | 7 | 0,56 | 0,17 | 4 |
| **38** | 334.37 | 0.34 | 2 | 7 | 0,53 | 0,18 | 5 |
| **39** | 228.25 | -0.86 | 1 | 6 | 0,80 | 0,20 | 1 |
| **40** | 270.32 | 0.71 | 1 | 6 | 0,85 | 0,31 | 3 |
| **41** | 270.32 | 0.71 | 1 | 6 | 0,85 | 0,31 | 3 |
| **42** | 242.27 | -0.08 | 1 | 6 | 0,82 | 0,27 | 2 |
| **43** | 272.3 | -0.7 | 2 | 7 | 0,83 | 0,33 | 3 |
| **44** | 270.28 | -1.33 | 1 | 7 | 0,83 | 0,25 | 3 |
| **45** | 286.32 | -0.55 | 1 | 7 | 0,85 | 0,23 | 4 |
| **46** | 268.27 | -1.77 | 1 | 7 | 0,67 | 0,25 | 3 |
| **47** | 330.38 | -0.81 | 2 | 8 | 0,87 | 0,27 | 6 |
| **48** | 270.28 | -1.36 | 1 | 7 | 0,83 | 0,33 | 2 |
| **49** | 350.37 | -0.42 | 1 | 8 | 0,53 | 0,18 | 5 |
| **50** | 320.34 | -0.39 | 1 | 7 | 0,50 | 0,19 | 4 |
| **51** | 396.44 | 1.24 | 1 | 7 | 0,36 | 0,14 | 5 |
| **52** | 306.31 | -0.13 | 1 | 7 | 0,47 | 0,20 | 4 |
| **53** | 256.3 | 0.27 | 1 | 6 | 0,83 | 0,25 | 3 |
| **54** | 480.64 | 5.46 | 0 | 8 | 0,88 | 0,15 | 16 |
| **55** | 560.72 | 6.4 | 0 | 9 | 0,71 | 0,10 | 19 |
| **56** | 238.24 | -0.9 | 0 | 6 | 0,82 | 0,36 | 0 |
| **57** | 318.32 | 0.04 | 0 | 7 | 0,50 | 0,19 | 3 |
| **58** | 381.38 | 2.35 | 1 | 7 | 0,33 | 0,19 | 4 |
| **59** | 240.26 | -0.24 | 1 | 6 | 0,82 | 0,36 | 1 |
| **60** | 279.38 | 3.56 | 0 | 2 | 0,26 | 0,05 | 4 |
| **61** | 309.4 | 3.57 | 0 | 3 | 0,30 | 0,10 | 5 |
| **62** | 219.28 | 1.61 | 1 | 3 | 0,38 | 0,15 | 3 |
| **63** | 293.4 | 3.96 | 0 | 2 | 0,30 | 0,10 | 4 |
| **64** | 433.58 | 4.66 | 0 | 6 | 0,64 | 0,12 | 10 |
| **65** | 435.6 | 6.29 | 0 | 4 | 0,46 | 0,11 | 8 |
| **66** | 433.58 | 4.66 | 0 | 6 | 0,64 | 0,16 | 10 |
| **67** | 335.39 | 1.78 | 1 | 6 | 0,50 | 0,22 | 7 |
| **68** | 335.39 | 2.33 | 1 | 6 | 0,50 | 0,17 | 7 |
| **69** | 303.35 | 2.24 | 0 | 5 | 0,47 | 0,29 | 3 |
| **70** | 427.45 | 0.87 | 3 | 8 | 0,43 | 0,26 | 6 |
| **71** | 277.27 | -2.21 | 3 | 8 | 0,91 | 0,45 | 3 |
| **72** | 277.27 | -2.21 | 3 | 8 | 0,91 | 0,45 | 3 |
| **73** | 459.49 | 0.41 | 4 | 9 | 0,46 | 0,25 | 8 |
| **74** | 459.49 | 0.41 | 4 | 9 | 0,46 | 0,25 | 8 |
| **75** | 457.47 | 1.44 | 3 | 9 | 0,46 | 0,29 | 6 |
| **76** | 427.45 | 2.05 | 2 | 8 | 0,43 | 0,26 | 5 |
| **77** | 369.37 | -0.18 | 3 | 9 | 0,59 | 0,35 | 6 |
| **78** | 389.42 | -0.37 | 3 | 9 | 0,63 | 0,38 | 4 |
| **79** | 427.45 | 2.05 | 2 | 8 | 0,43 | 0,26 | 5 |
| **80** | 397.42 | 2.12 | 1 | 7 | 0,41 | 0,23 | 4 |
| **81** | 189.21 | -0.17 | 1 | 5 | 0,88 | 0,38 | 3 |
| **82** | 189.21 | -0.17 | 1 | 5 | 0,88 | 0,38 | 3 |
| **83** | 411.45 | 3.27 | 0 | 7 | 0,39 | 0,17 | 6 |
| **84** | 411.45 | 3.27 | 0 | 7 | 0,39 | 0,17 | 6 |
| **85** | 397.42 | 2.95 | 1 | 7 | 0,36 | 0,18 | 5 |
| **86** | 397.42 | 2.95 | 1 | 7 | 0,36 | 0,18 | 5 |
| **87** | 367.4 | 2.93 | 1 | 6 | 0,33 | 0,14 | 4 |
| **88** | 381.42 | 3.26 | 0 | 6 | 0,36 | 0,14 | 5 |
| **89** | 365.38 | 3.08 | 0 | 6 | 0,24 | 0,10 | 5 |
| **90** | 367.4 | 2.82 | 0 | 6 | 0,33 | 0,10 | 5 |
| **91** | 379.41 | 3.51 | 0 | 6 | 0,27 | 0,14 | 5 |
| **92** | 353.37 | 2.5 | 1 | 6 | 0,30 | 0,10 | 4 |
| **93** | 205.21 | -1.22 | 2 | 6 | 0,88 | 0,38 | 4 |
| **94** | 265.3 | 1.11 | 1 | 5 | 0,50 | 0,21 | 4 |
| **95** | 323.34 | 1.65 | 0 | 7 | 0,50 | 0,19 | 6 |
| **96** | 291.3 | 1.89 | 0 | 6 | 0,33 | 0,13 | 5 |
| **97** | 405.44 | 2.39 | 0 | 8 | 0,57 | 0,24 | 8 |
| **98** | 363.36 | 1.33 | 0 | 8 | 0,50 | 0,22 | 8 |
| **99** | 363.36 | 1.33 | 0 | 8 | 0,50 | 0,22 | 8 |
| **100** | 321.33 | 0.68 | 1 | 7 | 0,50 | 0,25 | 6 |
| **101** | 335.31 | 0.64 | 1 | 8 | 0,44 | 0,25 | 6 |
| **102** | 391.42 | 1.95 | 0 | 8 | 0,55 | 0,20 | 8 |
| **103** | 391.42 | 1.95 | 0 | 8 | 0,55 | 0,20 | 8 |
| **104** | 747.88 | 5.35 | 4 | 14 | 0,50 | 0,15 | 17 |
| **105** | 428.52 | 0.94 | 4 | 10 | 0,80 | 0,20 | 11 |
| **106** | 527.65 | 1.32 | 5 | 12 | 0,80 | 0,20 | 13 |
| **107** | 696.83 | 2.97 | 5 | 16 | 0,73 | 0,18 | 18 |
| **108** | 512.55 | 3.3 | 0 | 8 | 0,30 | 0,17 | 8 |
| **109** | 512.55 | 3.3 | 0 | 8 | 0,30 | 0,17 | 8 |
| **110** | 440.49 | 2.88 | 0 | 6 | 0,26 | 0,15 | 5 |
| **111** | 440.49 | 2.88 | 0 | 6 | 0,26 | 0,15 | 5 |
| **112** | 440.49 | 3.31 | 0 | 6 | 0,26 | 0,15 | 4 |
| **113** | 440.49 | 3.31 | 0 | 6 | 0,26 | 0,15 | 4 |
| **114** | 456.49 | 2.92 | 0 | 7 | 0,26 | 0,15 | 5 |
| **115** | 456.49 | 2.92 | 0 | 7 | 0,26 | 0,15 | 5 |
| **116** | 471.46 | 2.77 | 0 | 9 | 0,23 | 0,15 | 5 |
| **117** | 471.46 | 2.77 | 0 | 9 | 0,23 | 0,15 | 5 |
| **118** | 485.49 | 3.14 | 0 | 9 | 0,26 | 0,15 | 5 |
| **119** | 485.49 | 3.14 | 0 | 9 | 0,26 | 0,15 | 5 |
| **120** | 470.52 | 3.28 | 0 | 7 | 0,29 | 0,14 | 5 |
| **121** | 470.52 | 3.28 | 0 | 7 | 0,29 | 0,14 | 5 |
| **122** | 519.39 | 4.0 | 0 | 6 | 0,26 | 0,15 | 4 |
| **123** | 519.39 | 4.0 | 0 | 6 | 0,26 | 0,15 | 4 |
| **124** | 420.5 | 3.13 | 0 | 6 | 0,44 | 0,16 | 5 |
| **125** | 396.39 | 1.39 | 1 | 8 | 0,33 | 0,14 | 5 |
| **126** | 397.38 | 1.96 | 0 | 8 | 0,33 | 0,14 | 6 |
| **127** | 452.54 | 3.17 | 2 | 7 | 0,46 | 0,19 | 9 |
| **128** | 418.53 | 2.9 | 2 | 7 | 0,65 | 0,22 | 9 |
| **129** | 436.5 | 2.15 | 1 | 7 | 0,44 | 0,20 | 6 |
| **130** | 410.46 | 1.85 | 2 | 7 | 0,39 | 0,22 | 7 |
| **131** | 465.54 | 1.41 | 1 | 8 | 0,46 | 0,15 | 7 |
| **132** | 452.54 | 3.17 | 2 | 7 | 0,46 | 0,19 | 9 |
| **133** | 362.38 | 1.04 | 2 | 8 | 0,50 | 0,22 | 6 |
| **134** | 362.38 | 1.04 | 2 | 8 | 0,50 | 0,22 | 6 |
| **135** | 330.38 | 1.11 | 0 | 6 | 0,56 | 0,17 | 3 |
| **136** | 362.42 | 1.15 | 2 | 7 | 0,58 | 0,21 | 7 |
| **137** | 362.42 | 1.15 | 2 | 7 | 0,58 | 0,21 | 7 |
| **138** | 362.42 | 1.15 | 2 | 7 | 0,58 | 0,21 | 7 |
| **139** | 452.54 | 2.83 | 1 | 7 | 0,46 | 0,15 | 9 |
| **140** | 409.52 | 1.93 | 1 | 8 | 0,86 | 0,19 | 8 |
| **141** | 457.56 | 2.64 | 1 | 8 | 0,64 | 0,16 | 8 |
| **142** | 409.52 | 1.93 | 1 | 8 | 0,86 | 0,19 | 8 |
| **143** | 457.56 | 2.64 | 1 | 8 | 0,64 | 0,16 | 8 |
| **144** | 340.41 | 0.68 | 1 | 7 | 0,88 | 0,24 | 5 |
| **145** | 388.41 | 1.23 | 0 | 8 | 0,55 | 0,20 | 5 |
| **146** | 368.42 | 1.36 | 0 | 8 | 0,83 | 0,22 | 7 |
| **147** | 368.42 | 1.36 | 0 | 8 | 0,83 | 0,22 | 7 |
| **148** | 400.47 | 1.3 | 2 | 9 | 0,84 | 0,26 | 10 |
| **149** | 388.41 | 1.23 | 0 | 8 | 0,55 | 0,20 | 5 |
| **150** | 368.42 | 1.26 | 0 | 8 | 0,83 | 0,28 | 6 |
| **151** | 368.42 | 1.26 | 0 | 8 | 0,83 | 0,28 | 6 |
| **152** | 368.42 | 1.26 | 0 | 8 | 0,83 | 0,22 | 6 |
| **153** | 368.42 | 1.26 | 0 | 8 | 0,83 | 0,22 | 6 |
| **154** | 354.4 | 0.9 | 0 | 8 | 0,82 | 0,24 | 5 |
| **155** | 354.4 | 0.93 | 1 | 8 | 0,82 | 0,24 | 5 |
| **156** | 354.4 | 0.93 | 1 | 8 | 0,82 | 0,24 | 5 |
| **157** | 354.4 | 0.93 | 1 | 8 | 0,82 | 0,24 | 5 |
| **158** | 402.44 | 1.52 | 0 | 8 | 0,57 | 0,19 | 6 |
| **159** | 354.4 | 0.9 | 0 | 8 | 0,82 | 0,24 | 5 |
| **160** | 402.44 | 1.52 | 0 | 8 | 0,57 | 0,19 | 6 |
| **161** | 330.38 | 1.11 | 0 | 6 | 0,56 | 0,17 | 3 |
| **162** | 340.41 | 0.68 | 1 | 7 | 0,88 | 0,24 | 5 |
| **163** | 362.42 | 1.15 | 2 | 7 | 0,58 | 0,21 | 7 |
| **164** | 334.32 | -0.42 | 2 | 8 | 0,44 | 0,19 | 6 |
| **165** | 286.33 | 0.73 | 1 | 5 | 0,44 | 0,19 | 4 |
| **166** | 390.43 | 2.22 | 1 | 6 | 0,30 | 0,17 | 6 |
| **167** | 293.34 | 1.25 | 0 | 5 | 0,43 | 0,21 | 4 |
| **168** | 383.46 | 3.28 | 0 | 5 | 0,33 | 0,19 | 6 |
| **169** | 509.62 | 2.93 | 0 | 8 | 0,44 | 0,15 | 7 |
| **170** | 509.62 | 2.93 | 0 | 8 | 0,44 | 0,15 | 7 |
| **171** | 383.46 | 3.28 | 0 | 5 | 0,33 | 0,19 | 6 |
| **172** | 348.42 | 0.49 | 0 | 6 | 0,53 | 0,18 | 3 |
| **173** | 438.54 | 2.51 | 0 | 6 | 0,42 | 0,17 | 5 |
| **174** | 419.49 | 0.9 | 0 | 8 | 0,55 | 0,15 | 5 |
| **175** | 438.54 | 2.51 | 0 | 6 | 0,42 | 0,17 | 5 |
| **176** | 263.29 | 1.26 | 0 | 5 | 0,43 | 0,14 | 4 |
| **177** | 249.26 | 0.83 | 0 | 5 | 0,38 | 0,08 | 4 |
| **178** | 355.38 | 2.33 | 0 | 6 | 0,30 | 0,10 | 6 |
| **179** | 426.51 | 3.49 | 0 | 5 | 0,26 | 0,11 | 5 |
| **180** | 456.53 | 3.46 | 0 | 6 | 0,29 | 0,11 | 6 |
| **181** | 369.41 | 2.52 | 0 | 6 | 0,33 | 0,10 | 6 |
| **182** | 442.51 | 3.46 | 0 | 6 | 0,26 | 0,07 | 5 |
| **183** | 412.48 | 3.06 | 0 | 5 | 0,23 | 0,08 | 5 |

Table S4: Fsp^3^ and FC* data for a reference set of BB drugs^[[4]](#endnote-3)^

| **Drug** | **Fsp3** | **FC*** |  | **Drug** | **Fsp3** | **FC*** |
| --- | --- | --- | --- | --- | --- | --- |
| **Lipitor** | 0,27 | 0,06 |  | **Topamax** | 1,00 | 0,33 |
| **Nexium** | 0,29 | 0,00 |  | **Toprol** | 0,60 | 0,00 |
| **Prevacid** | 0,25 | 0,00 |  | **Zetia** | 0,21 | 0,13 |
| **Flonase** | 0,72 | 0,36 |  | **Fosamax** | 1,00 | 0,00 |
| **Serevent** | 0,52 | 0,00 |  | **Abilify** | 0,43 | 0,00 |
| **Singulair** | 0,29 | 0,03 |  | **Levaquin** | 0,39 | 0,06 |
| **Effexor** | 0,65 | 0,00 |  | **Lamictal** | 0,00 | 0,00 |
| **Plavix** | 0,31 | 0,06 |  | **Celebrex** | 0,12 | 0,00 |
| **Zocor** | 0,76 | 0,28 |  | **Benazepril** | 0,38 | 0,08 |
| **Norvasc** | 0,40 | 0,00 |  | **Zyrtec** | 0,38 | 0,00 |
| **Lexapro** | 0,35 | 0,05 |  | **Coreg** | 0,25 | 0,00 |
| **Seroquel** | 0,38 | 0,00 |  | **Valtrex** | 0,54 | 0,08 |
| **Protonix** | 0,25 | 0,00 |  | **Adderall** | 0,33 | 0,00 |
| **Ambien** | 0,26 | 0,00 |  | **Aciphex** | 0,33 | 0,00 |
| **Actos** | 0,32 | 0,00 |  | **Cymbalta** | 0,22 | 0,06 |
| **Zoloft** | 0,29 | 0,12 |  | **Crestor** | 0,41 | 0,09 |
| **Wellbutrin** | 0,46 | 0,00 |  | **Diovan** | 0,38 | 0,04 |
| **Avandia** | 0,28 | 0,06 |  | **Tricor** | 0,30 | 0,00 |
| **Risperdal** | 0,52 | 0,00 |  | **Concerta** | 0,50 | 0,00 |
| **Zyprexa** | 0,35 | 0,00 |  | **Imitrex** | 0,43 | 0,00 |

1. * Corresponding authors. Tel.: +39-055-457-3507; fax: +39-055-457-4913; e-mail: [elena.lenci@unifi.it](mailto:elena.lenci@unifi.it); andrea.trabocchi@unifi.it [↑](#footnote-ref-1)
2. **References**

   Dar’in, D., Bakulina, O., Chizhova, M., and Krasavin, M. (2015). New heterocyclic product space for the Castagnoli−Cushman three-component reaction. *Org. Lett.* 17, 3930−3933. doi: 10.1021/acs.orglett.5b02014. [↑](#endnote-ref-1)
3. Brown, G. R., Foubister, A. J., and Wright, B. (1985). Chiral synthesis of 3-substituted morpholines via serine enantiomers and reductions of 5-oxomorpholine-3-carboxylates. *J. Chem. Soc., Perkin Trans.* 1, 2577 – 2580. doi: 10.1039/P19850002577 [↑](#endnote-ref-2)
4. Bauer, R. A., Wurst, J. M., and Tan, D. S. (2010). Expanding the range of 'druggable' targets with natural product-based libraries: an academic perspective. *Curr. Opin. Chem. Biol.* 14, 308-314. doi: 10.1016/j.cbpa.2010.02.001 [↑](#endnote-ref-3)
